# Guide to lower extremity radiologic measurements: part 3 ankle and foot

**DOI:** 10.1007/s00256-026-05188-1

**Published:** 2026-03-30

**Authors:** Allison M. Crone, Imran M. Omar, Allison M. Bronson, Muhammad Y. Mutawakkil, Ryan S. Selley, Jennifer S. Weaver, Andrea S. Klauser, Mihra S. Taljanovic

**Affiliations:** 1 Department of Radiology and Imaging Sciences, Indiana University School of Medicine, 714 North Senate Ave., Suite 200, Indianapolis, IN 46202 USA; 2 Department of Radiology, Northwestern University, Feinberg School of Medicine, 676 North Saint Clair St., Suite 800, Chicago, IL 60611 USA; 3 Department of Orthopaedic Surgery, Northwestern University, Feinberg School of Medicine, 676 North Saint Clair St., Suite 1350, Chicago, IL 60611 USA; 4 Department of Radiology, University of Texas at San Antonio, 7703 Floyd Curl Drive, San Antonio, TX 78229 USA; 5 Department of Radiology, Innsbruck Medical University, Privatpraxis Bruneckerstr 2E/5. Stock, Innsbruck, Austria; 6 Departments of Radiology and Imaging Sciences and Orthopaedic Surgery, The University of Arizona, College of Medicine, 1501 North Campbell Ave., Tucson, AZ 85724 USA; 7 Department of Radiology, University of New Mexico, MSC 10 5530, 1 University of New Mexico, Albuquerque, NM 87131 USA

**Keywords:** Ankle joint, Foot anatomy, Radiography, Computed tomography, Magnetic resonance imaging, Weight-bearing

## Abstract

The ankle and foot rely on a complex, coordinated interaction of numerous small bones and supporting soft tissues to produce efficient locomotion and weight bearing. Subtle osseous malalignments or insufficiency of tendons and ligaments can result in pain, instability and arthropathy. Although gross malalignment can be seen qualitatively, detecting subtle malalignment often requires standardized measurement on imaging studies with prescribed techniques. Moreover, stress imaging techniques, such as weightbearing or angular stress, are more often used in ankle/foot imaging compared with other lower extremity joints, which results in an added layer of complexity when performing measurements. In the ankle/hindfoot, quantitative assessment is most commonly used to detect and/or characterize ankle instability, calcaneal fractures and posterior tibialis tendon dysfunction. Furthermore, quantitative assessments of ankle arthroplasties may help diagnose device failure. In the midfoot, assessment of the tarsometatarsal joints can help detect subtle Lisfranc instability, while in the forefoot, quantitative characterization of hallux valgus is often used to guide surgical management. This review article is the last of a three-part series discussing measurements of the lower extremities and focuses on common measurements used in the foot and ankle, grouped by pathology, with attention to the imaging study of choice for each measurement, as well as the appropriate technique of measurement.

## Introduction

Many musculoskeletal disorders in the foot and ankle can be evaluated via qualitative assessment of diagnostic images; however, there are some cases in which quantitative measurements are necessary to make the diagnosis or guide management. For example, foot and ankle trauma leading to ligament insufficiency, calcaneal fractures leading to subtalar pathology, alignment issues stemming from loss of the medial midfoot arch and hallux valgus commonly require measurements to help determine the course of treatment and whether surgery is indicated. When making measurements on diagnostic imaging of the ankle and foot, it is critical to utilize measurements that have well-established normal values and protocols to standardize assessment and avoid inaccuracies that may lead to errors in management. This review article will discuss the method of measurement, normal values, and implications of abnormal values for many of the commonly used measurements in the ankle and foot.

## Ankle/hindfoot

### Imaging modalities

#### Radiography

Radiography is often the first imaging modality used to evaluate ankle pain and/or injury. A typical ankle radiographic series includes anteroposterior (AP), mortise, and lateral views. On the AP radiograph, the X-ray beam should be centered over the talar dome or tibiotalar joint centered between the medial and lateral malleoli, and the fibula should slightly overlap the lateral talar dome. The mortise radiograph is also centered either at the talar dome or the tibiotalar joint. However, the ankle is internally rotated by 15°–20° to bring the talar dome and ankle mortise in profile. In this view, there is often slight overlap between the distal tibia and fibula at the syndesmosis, and the lower limb may need to be further internally rotated to assess the syndesmosis in profile if needed. Finally, the lateral radiograph is centered at the talar dome or tibiotalar joint, bringing the tibiotalar joint in sharp profile. Because proximal fifth metatarsal base fractures are common, many institutions include this in the imaged field-of-view on lateral ankle radiographs. Well-positioned radiographs are vital in orthopaedics since they allow standardized measurements, such as the assessment of the talar tilt angle or anterior translation, which can be compared prior to and after any intervention and followed with serial imaging. Weightbearing is the most common stress and allows assessment of alignment with physiologic load-bearing. Stress radiographs of the ankle can also be helpful to assess ligamentous and/or syndesmotic integrity. These include AP varus stress (inversion), AP valgus stress (external rotation), and lateral anterior drawer radiographs. Stress can be applied manually (either via hand or with various devices available for the purpose of stress radiograph positioning) or with gravity [1]. Gravity-stress radiography is as reliable as manual stress imaging in detecting deltoid ligament insufficiency or syndesmotic widening; avoids exposing the clinician to ionizing radiation laterally; and is more comfortable for patients since it requires less force [2, 3]. Abnormalities in measurements obtained on these various stress views may suggest underlying injury to the soft tissue stabilizers, as discussed subsequently.

#### Cross-sectional imaging

Computed tomography (CT) and magnetic resonance imaging (MRI) are frequently utilized to evaluate the ankle and foot. CT provides precise osseous detail and is often used for fracture evaluation, pre-operative planning, and postoperative/hardware assessment. It allows for multiplanar and 3-dimensional reformats, which are particularly helpful for pre-operative planning. Compared with radiographs, CT provides better 3-dimensional evaluation and is not limited by projection or overlapping structures.

Until recently, CT imaging could not be performed with weightbearing, and thus, was unable to provide an accurate representation of the physiologic alignment of a patient’s foot. However, new advances in cone-beam CT (CBCT) have led to the development of weightbearing CT scanners, in which a large detector array and a pyramid-shaped X-ray beam (“cone” beam) allow CT data to be obtained with a single rotation around the patient [4]. With this configuration. patients can stand for the CT acquisition, placing their feet in more physiologic positions, resulting in more accurate measurements and precise surgical planning [5]. Compared with whole-body multidetector CT (MDCT) systems, CBCT has a similar geometric accuracy, and may even be slightly better than MDCT for small fields-of-view [6]. In addition to weightbearing acquisition, CBCT is particularly helpful in imaging the foot and ankle since it allows exquisite bony trabecular detail with greatly reduced radiation doses, and has metal artifact reduction capability; these factors make CBCT attractive for serial imaging. On the other hand, CBCT images often have lower signal-to-noise, which can greatly limit soft tissue assessments, and this technology is not widely available.

Several published articles have compared some of the common measurements obtained from weightbearing radiography with those from weightbearing CBCT with promising results. However, further work must be performed to verify the accuracy of additional measurements obtained from weightbearing CBCT that have not already been compared with radiography.

Compared with other imaging modalities, MRI is the test of choice for evaluating tendinous and ligamentous soft tissue structures of the ankle, though this modality is limited since it cannot commonly be performed with weightbearing and is therefore not generally used to make definitive ankle measurements. In clinical practice, some radiographic measurements can be applied to MRI to help suggest certain pathologies; however, the abnormalities must be confirmed with weightbearing radiography or CT to establish the diagnosis.

### Measurements (Table 1)

**Table 1:** Commonly used orthopaedic measurements in assessment of the ankle

Radiologic measurement	Definition	Imaging modality of choice	Normal value(s)/normal range	Abnormal value(s)/clinical significance
Talar tilt angle	Angle formed by lines drawn along the tibial plafond and talar dome (Fig. 1)	AP or mortise ankle XR	< 2° varus angulation < 5° with stress	≥ 2° varus angulation or ≥ 5° with stress indicates lateral ligamentous complex insufficiency
Medial clear space (tibiotalar space)	Horizontal distance from the medial talar articular surface to the medial malleolus articular surface (Fig. 2)	Mortise XR	< 4–5 mm	> 5 mm on nonstress, or increase of > 2 mm with stress compared with nonstress indicates deltoid insufficiency
Talocrural angle	Angle formed either by a line tangent to the tibial plafond or along the tibial long axis, perpendicular to plafond, and a line connecting tips of the malleoli (Fig. 3)	Mortise XR	83° ± 4° if using tibial long axis 8°–15° if using the tibial plafond	< 2°–4° difference compared with contralateral side to see if there is excessive fibular shortening
Anterior talar translation	Shortest distance between the posterior lip of the tibial plafond and the posterior talar dome articular surface (Fig. 4)	Lateral ankle XR	< 4 mm in neutral < 6 mm with stress	≥ 4 mm at rest or ≥ 6 mm with anterior stress indicates anterior talofibular ligament insufficiency
Tibiofibular clear space	Horizontal distance from the tibial incisura fibularis to the medial margin of the distal fibula (Fig. 5)	Mortise or AP ankle XR	< 6 mm	≥ 6 mm indicates syndesmotic ligament insufficiency
Tibiofibular overlap	Horizontal distance between the medial contour of the fibula and the lateral contour of the anterior tibial tubercle (Fig. 6)	AP or mortise ankle XR	> 6 mm on AP XR > 1 mm on mortise XR	≤ 6 mm on AP XR or ≤ 1 mm on mortise XR can indicate syndesmotic instability
Böhler’s angle	Angle formed by a line connecting the anterior calcaneal process and superior margin of the posterior calcaneal facet, and a line connecting the posterior calcaneal facet and superior margin of the calcaneal tuberosity (Fig. 7)	Lateral foot XR	25°–40°	< 25° indicates posterior calcaneal facet depression
Critical angle of Gissane	Angle formed by lines along the superior surface of anterior calcaneal process and posterior calcaneal facet (Fig. 8)	Lateral foot XR	120°–145°	> 145° indicates calcaneal depression at subtalar joint
Tibiocalcaneal (hindfoot valgus) angle	Angle formed between the tibial long axis and the calcaneal body axis on coronal imaging (Fig. 9)	WB hindfoot alignment XR or coronal reformatted CBCT	≤ 6°	**·** ≥ 7° hindfoot valgus
Lateral talocalcaneal angle	Angle formed by a line along the talar neck long axis and a line along the inferior calcaneal surface (Fig. 10)	Lateral foot XR	25°–40°	**·** < 25°—pes cavus **·** > 40°- pes planus and hindfoot valgus
Meary (lateral talus – first metatarsal) angle	Angle formed between a line along the talar head/neck long axis, and the first metatarsal anatomic axis (Fig. 11)	Lateral foot XR	0° ± 4°	**·**5°–15° mild pes planus **·**16°–30° moderate pes planus **·** > 30° severe pes planus
Calcaneal pitch/inclination angle	Angle formed by a horizontal line along the plantar surface of the foot and a line along the inferior surface of the calcaneus (Fig. 12)	Lateral foot XR	20°–30°	**·** < 20° suggests pes planus **·** > 30° suggests pes cavus
Talonavicular uncoverage percentage	Talar head articular surface that is uncovered by the navicular divided by the total length of the talar articular surface × 100% (Fig. 13)	DP foot XR	≤ 30%	> 30% stage IIB acquired flatfoot deformity
AP talocalcaneal (Kite) angle	Angle formed by line along the long axis of the talar neck, and a line along the lateral border of the calcaneus (Fig. 14)	DP foot XR	25°–40°	**·** < 25° suggests hindfoot varus **·** > 40° suggests hindfoot valgus
Talonavicular coverage angle	Angle formed by a line connecting the medial and lateral margins of the talar articular surface and a line connecting the medial and lateral margins of the navicular articular surface (Fig. 15)	DP foot XR	2°–20°	> 20° associated with abnormal forefoot abduction and flatfoot
Talonavicular incongruency angle	Angle formed by a line connecting the lateral talar and navicular articular surfaces, and a line connecting the lateral margin of the talar neck at its narrowest point and the lateral talar articular surface (Fig. 16)	DP foot XR	−5°–20°	> 45° or > 50° stage IIB flatfoot deformity
AP talo-first metatarsal angle	Angle formed between lines along the talar neck long axis and first metatarsal anatomic axis (Fig. 17)	DP foot XR	7° ± 4°	> 11° excessive forefoot abduction

#### Ankle instability

##### Talar tilt angle

**Fig. 1:**
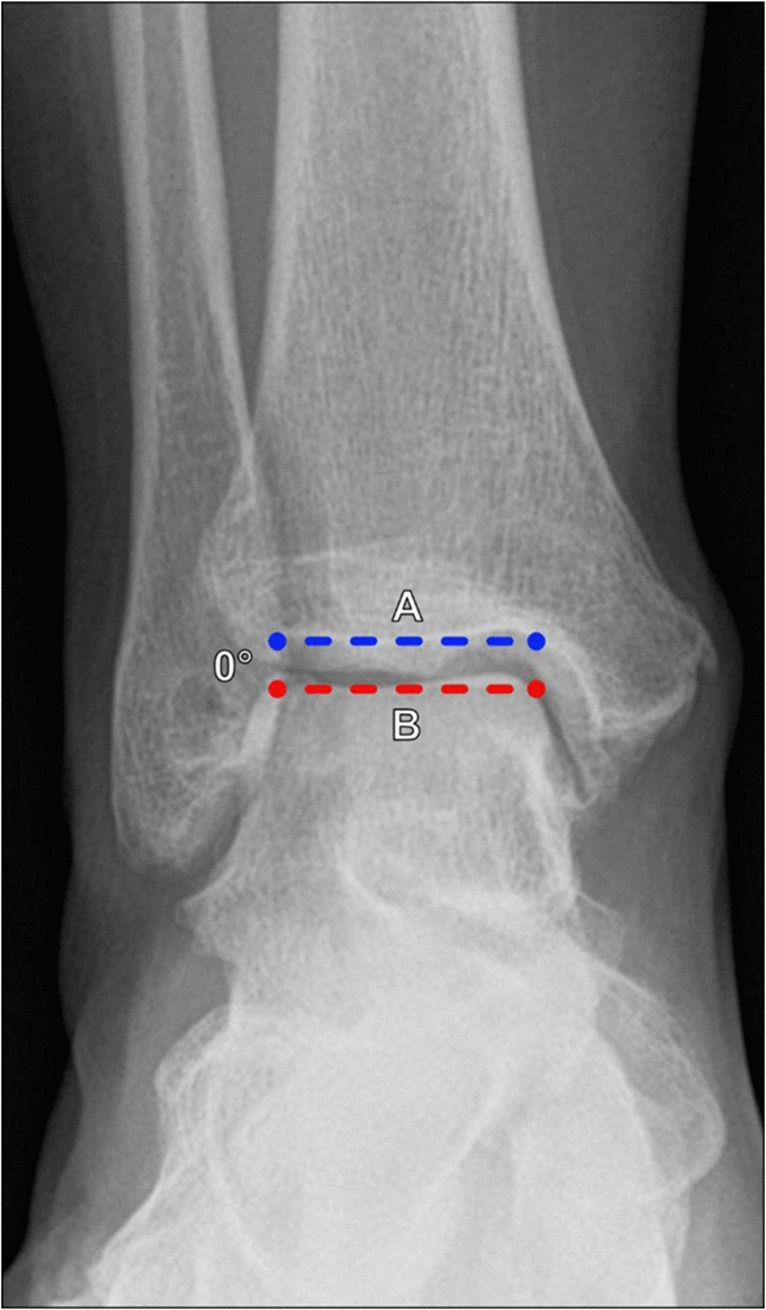
Measurement of the talar tilt angle on an AP radiograph of the ankle. The angle is formed from a line tangent to the tibial plafond (A, blue line) and a line along the talar dome (B, red line). In practice, accepted normal values in neutral position are < 2° of varus angulation; with supination/inversion stress the angle should measure < 5°. This measurement reflects the integrity of the calcaneofibular and anterior talofibular ligaments

The talar tilt angle is a measure of the lateral opening of the tibiotalar joint and generally reflects the integrity of the calcaneofibular and anterior talofibular ligaments. Measured on the AP or mortise view ankle radiographs, it is defined as the angle formed between lines drawn along the tibial plafond and talar dome (Fig. 1). There is wide variability in reported normal values in the literature with stress measurements ranging from 0° to 27° [7]. In practice, accepted normal values in neutral position are < 2° of varus angulation [8], and with supination/inversion stress the angle should measure < 5° [9].

##### Medial clear space (tibiotalar space)

**Fig. 2:**
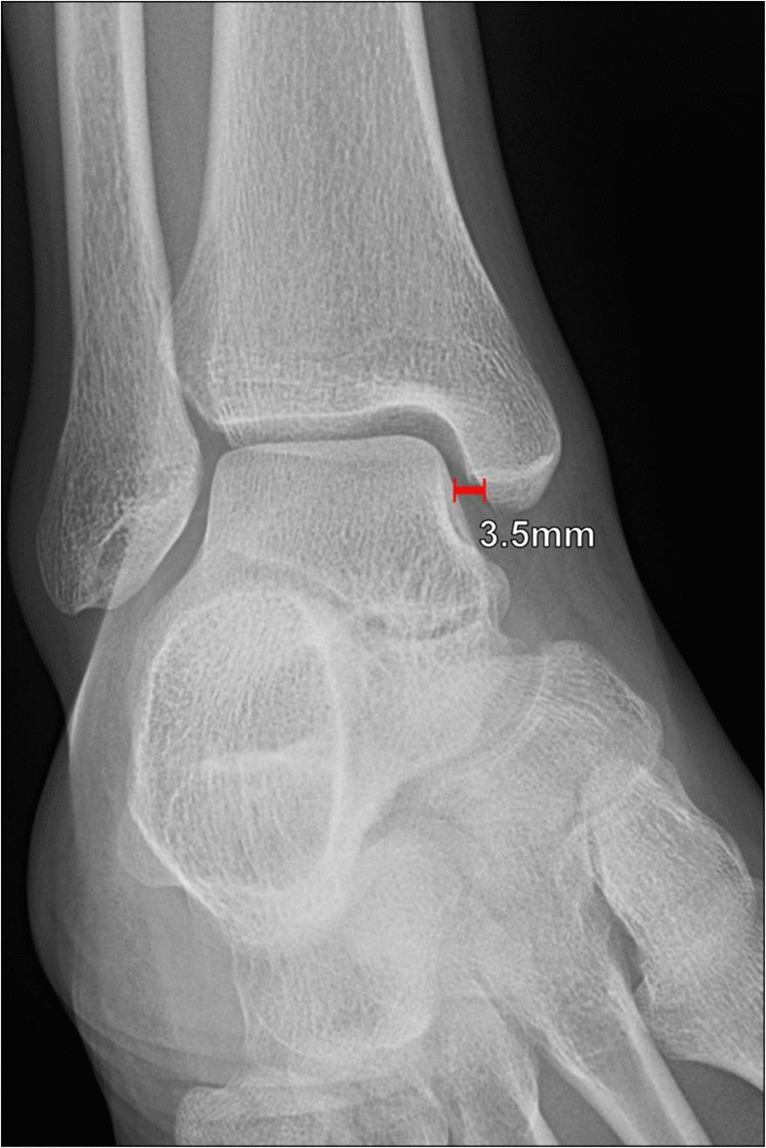
Measurement of medial clear space on mortise view of the ankle. This represents the horizontal distance between the lateral margin of the medial malleolus and the medial margin of the talus. It should normally measure < 4–5 mm and an increase in the medial clear space > 2 mm on stress radiographs relative to baseline neutral radiographs is associated with deep deltoid ligamentous injury and syndesmotic injury

The medial clear space is best assessed on the mortise radiograph and represents the horizontal linear distance between the lateral margin of the medial malleolar articular surface and the medial margin of the talar articular surface (Fig. 2). Multiple studies have validated this measurement, with reported normal values of < 4–5 mm [1, 10]. While malalignment of the ankle mortise and medial clear space widening is often evident on neutral radiographs, external rotation stress radiographs may be helpful to elicit medial clear space widening in more subtle cases [1]; an increase in the medial clear space > 2 mm on stress radiographs relative to baseline neutral radiographs has been correlated with deep deltoid ligamentous injury and syndesmotic injury [2, 10].

#### Less commonly used measurements to assess ankle stability

##### Talocrural angle

**Fig. 3:**
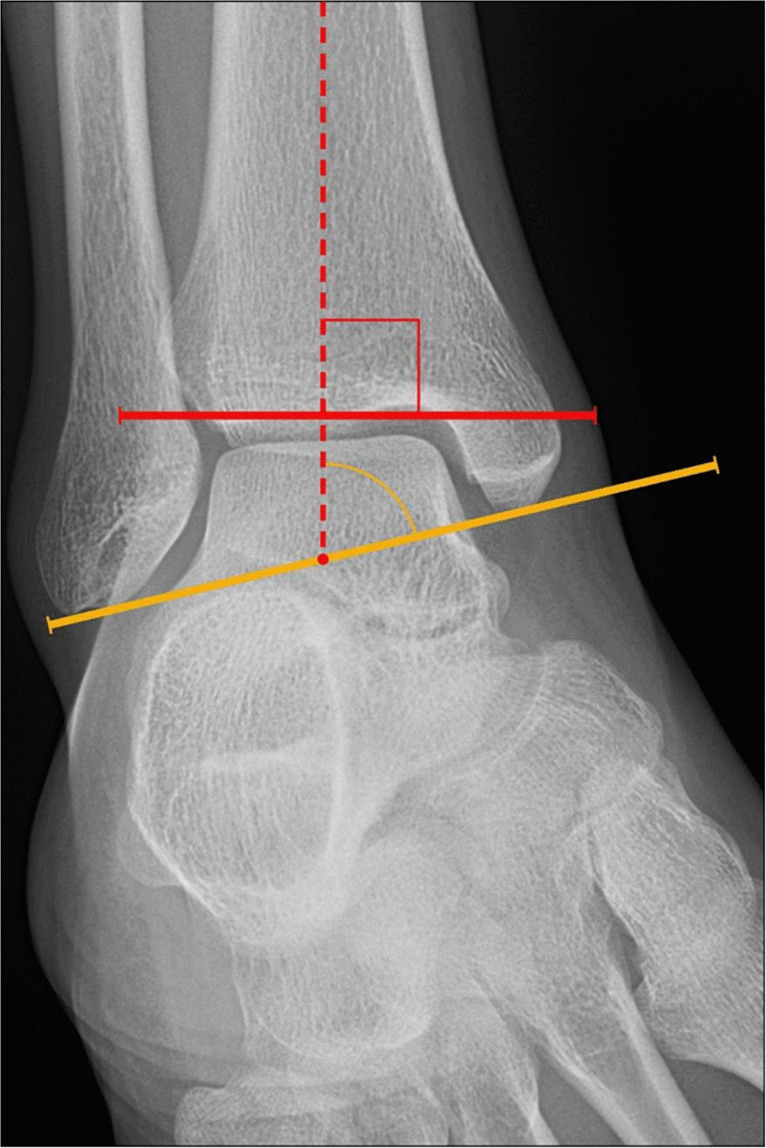
Measurement of the talocrural angle on ankle mortise radiograph. The angle (yellow angle notation) is created from a line connecting the tips of the medial and lateral malleoli (yellow line) and the tibial axis (dashed line), which is perpendicular to the tibial plafond articular surface (solid red line). Comparison with the contralateral side if unaffected may help detect subtle fibular shortening. Normal values are 83° ± 4° with < 2°–4° difference with the contralateral side. When using the tibial axis for the frame of reference, increased angle measurements indicate tibial shortening. Alternatively, the angle between a line tangent to the tibial plafond (solid red line) and a line connecting the tips of the medial and lateral malleoli (solid yellow line) may be taken, with normal values of 8°–15°. When using the tibial plafond as the reference line, decreased angles suggest lateral malleolar shortening

The talocrural angle is determined on the mortise radiograph and assesses the relative length of the fibula, which can become shortened in the setting of fracture (Fig. 3). Restoring fibular length is paramount in successfully treating ankle fractures and preventing post-traumatic arthropathy. This angle can be assessed in two ways. Either it can be measured by taking a line perpendicular to the tibial plafond articular surface and a second line connecting the tips of the medial and lateral malleoli, or it can be taken from a line along the tibial plafond articular surface and a second line connecting the tips of the medial and lateral malleoli. If the first method of reporting is performed, the talocrural angle should measure 83° ± 4°, and increased angles indicate fibular shortening. On the other hand, if the second method is used, the angle should measure 8°–15°, and decreased angles suggest fibular shortening. There should be < 2°–4° difference with the contralateral side. This measurement can be affected by limb rotation or X-ray beam divergence [11, 12].

##### Anterior talar translation

**Fig. 4:**
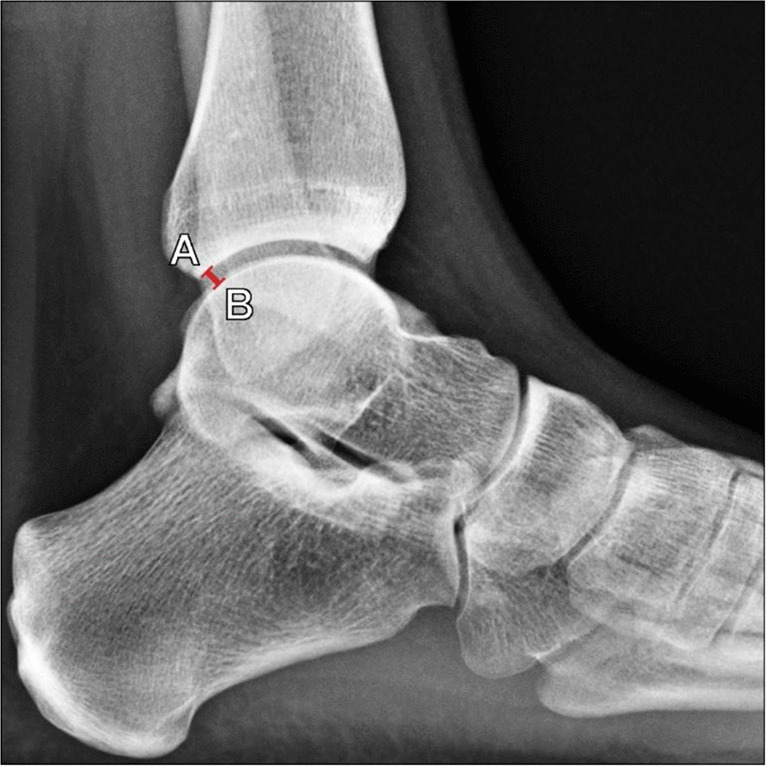
Measurement of the anterior talar translation on a lateral weightbearing radiograph of the ankle. The most reliable method for evaluation of anterior talar translation measures the shortest distance between the posterior lip of the tibial plafond (A) and the posterior talar dome articular surface (B). Normal values in neutral position are < 4 mm, and < 6 mm with anterior drawer stress. An increased anterior talar translation distance indicates anterior talofibular ligament insufficiency

The anterior talar translation measurement is measured on lateral ankle radiographs and is helpful in evaluating anterior talofibular ligament integrity (Fig. 4). It measures the shortest distance between the posterior lip of the tibial plafond and the posterior talar dome. This measurement has been described under manual stress and with various mechanical devices, with variable agreement between methods and observers and a wide range of reported normal and abnormal values. Using this method of measurement, normal values in the neutral position are < 4 mm, and anterior talar displacement ≥ 4 mm suggests anterior talofibular ligament insufficiency. With anterior drawer stress, it should measure < 6 mm [13–16].

#### Syndesmosis

Injuries to the distal tibiofibular syndesmosis can have important implications for ankle stability. As the talus is typically more tightly aligned with the lateral malleolus due to the strong lateral stabilizers, even slight widening of the tibiofibular interval can lead to lateral talar shift, resulting in decreased tibiotalar contact and ankle joint instability [17]. Syndesmotic injury may occur as an isolated soft tissue injury but often occurs in combination with lateral and/or medial malleolar fractures and deltoid ligament insufficiency [18]. The radiographic relationship between the distal tibia and fibula, including tibiofibular clear space widening and decreased tibiofibular overlap, can suggest syndesmotic injury. Although syndesmotic injuries can be assessed on AP views of the ankle, the mortise view is considered more reliable clinically since it more directly visualizes the joint.

It should be noted that some studies have called into question the accuracy of the tibiofibular clear space and tibiofibular overlap in predicting syndesmotic injury, including a study by Shah et al. which indicated that approximately 5–10% of normal patients may demonstrate variant tibiofibular clear space widening and diminished tibiofibular overlap in the absence of syndesmotic injury [19]. Comparison with the normal contralateral side and interpreting radiographs within the context of the Lauge-Hansen classification of ankle injury mechanisms [19, 20] have been shown to increase diagnostic accuracy.

##### Tibiofibular clear space

**Fig. 5:**
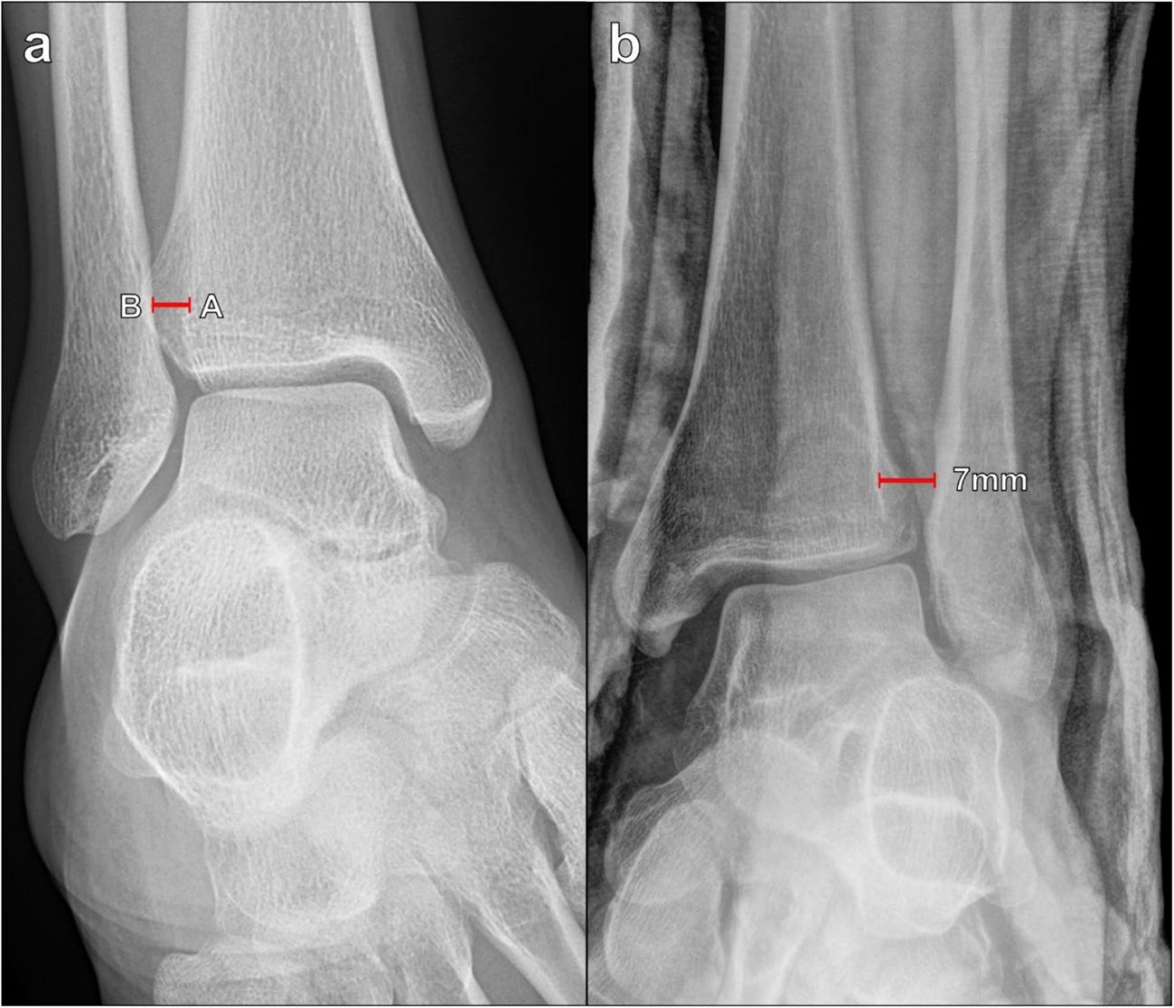
Measurement of the tibiofibular clear space on mortise radiograph of the ankle. **a** The measurement is made between the distal tibial incisura fibularis (A) to the medial margin of the distal fibula (B) at a level 1 cm superior to the tibial plafond. The normal tibiofibular clear space measures < 6 mm, and a measurement ≥ 6 mm indicates syndesmotic disruption. **b** Widening of the tibiofibular clear space on mortise radiograph of the ankle in a 20-year-old male patient after a fall. The tibiofibular distance measures 7 mm, and there is widening of the medial tibiotalar clear space, indicating deltoid ligament insufficiency as well

The tibiofibular clear space is the radiographic depiction of the syndesmosis, and widening of this space indicates tibiofibular ligament insufficiency. It can be measured on AP or mortise radiographs of the ankle. The measurement is made 1 cm superior to the tibial plafond, extending a horizontal line from the distal tibial incisura fibularis to the medial margin of the distal fibula (Fig. 5). The normal tibiofibular clear space measures < 6 mm and commonly measures 4–5 mm on both AP and mortise views; a measurement ≥ 6 mm indicates syndesmotic disruption [21].

##### Tibiofibular overlap

**Fig. 6:**
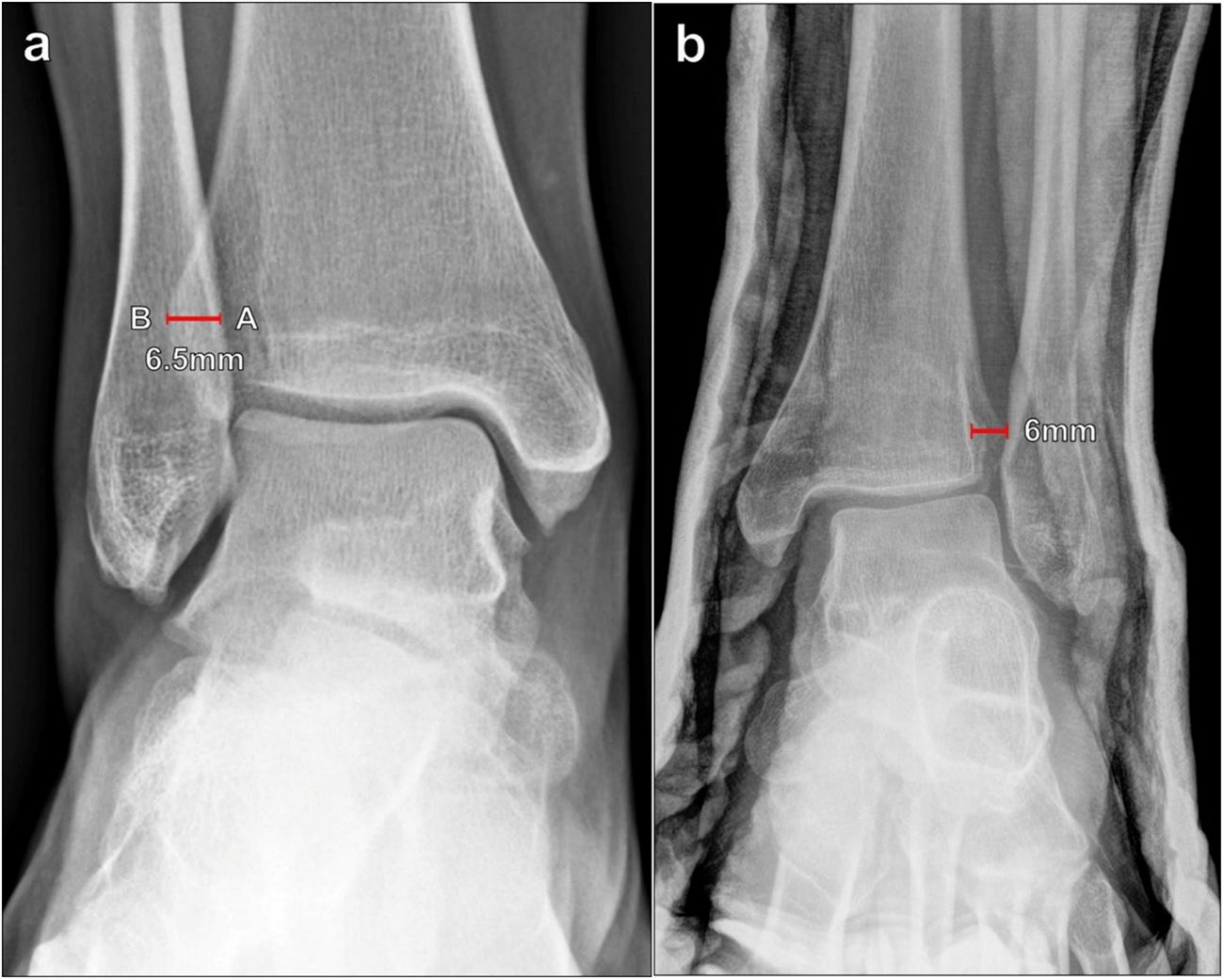
Measurement of tibiofibular overlap on AP radiograph of the ankle. **a** The horizontal distance between the medial margin of the distal fibula (A) and the lateral margin of the tibial tubercle (B) is measured 1 cm proximal to the tibial plafond. The tibiofibular overlap should be > 6 mm on the AP view and > 1 mm on the mortise view. The absence of tibiofibular overlap in conjunction with tibiofibular and medial tibiotalar diastasis suggests syndesmotic instability. **b** Absent tibiofibular overlap on AP radiograph of the ankle on the same patient seen on Fig. 5b, indicating syndesmotic insufficiency. Even on the AP radiograph, the tibiofibular clear space measures 6 mm, which also indicates syndesmotic disruption

Tibiofibular overlap can also be measured on AP or mortise views of the ankle. The tibiofibular overlap is measured 1 cm superior to the tibial plafond by taking the horizontal distance between the medial contour of the fibula and the lateral contour of the anterior tibial tubercle (Fig. 6). This measurement is highly dependent on patient rotation and should be interpreted along with the tibiofibular clear space distance. Harper et al. reported that the tibiofibular overlap should be > 6 mm or approximately 42% of the fibular width on the AP view and > 1 mm on the mortise view [21]. In general, there is typically > 0 mm of tibiofibular overlap regardless of rotation [22], though Shah et al. did demonstrate 4.9% of normal patients without overlap, which may reflect variant anatomy [19]. However, absent tibiofibular overlap associated with tibiofibular and medial tibiotalar diastasis highly suggests syndesmotic instability.

#### Calcaneal fractures

Most calcaneal fractures are the result of high energy trauma with significant axial load resulting in talar impact onto the calcaneus, typically resulting in collapse of the calcaneus and subtalar joint depression. There are several tools and classification systems for diagnostic and prognostic evaluation of calcaneal fractures. The two most commonly reported measurements are Böhler’s angle and the critical angle of Gissane, which are important metrics for determining the degree of injury and presurgical planning. Of these two, restoration of Böhler’s angle correlates better with functional outcomes.

##### Böhler’s angle

**Fig. 7:**
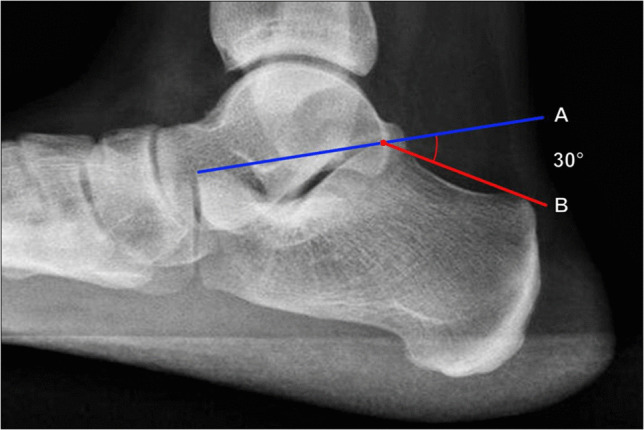
Measurement of Bohler’s angle on weightbearing lateral radiograph of the foot. The angle is formed between a line extending from the tip of the anterior calcaneal process to the superior margin of the posterior calcaneal facet (A, blue line) and a line extending from the posterior calcaneal facet to the superior margin of the calcaneal tuberosity (B, red line). Generally accepted normal values range from 25° to 40°, and values < 25° suggest depression of the calcaneal articular surface with more severe deformities

Initially described in 1931 by Böhler [23], this is the most commonly used angle to evaluate the severity of calcaneal fractures. Proper measurement technique is performed on lateral foot radiographs (Fig. 7), though lateral ankle radiographs can be used for screening purposes. The angle is formed by the intersection of two lines: a line connecting the tip of the anterior calcaneal process and the superior margin of the posterior calcaneal facet; and a line connecting the superior margin of the posterior calcaneal facet and the superior margin of the calcaneal tuberosity [1].

As originally described by Böhler, normal values range from 30° to 35° with a cutoff of < 28° considered pathologic [23]. However, many subsequent studies have reported different normal values for the angle, particularly with differences between ethnicities. In practice, generally accepted normal values range from 25° to 40° [24]. Lower values indicate more severe fractures with greater posterior calcaneal facet depression, and are associated with poorer outcomes. However, a normal Böhler’s angle does not exclude a non-displaced calcaneal fracture. Additionally, while restoration of Böhler’s angle is generally felt to improve postoperative function, studies have shown mixed results in terms of the prognostic value of the measurement on postoperative outcomes [25, 26].

##### Critical angle of Gissane

**Fig. 8:**
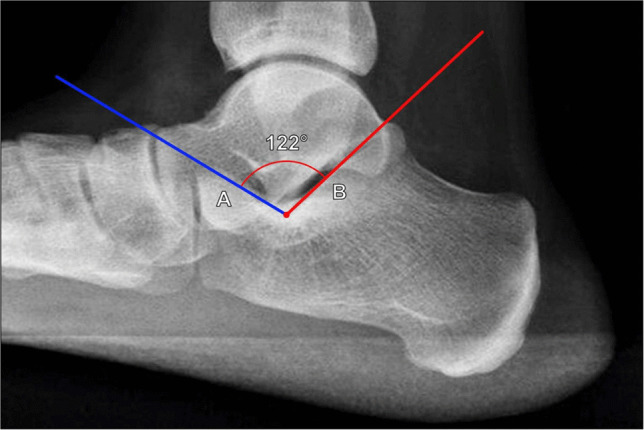
Measurement of the critical angle of Gissane on lateral weightbearing radiograph of the foot. The angle is formed by lines drawn along the superior surface of the anterior calcaneal process (A, blue line) and along the posterior calcaneal facet (B, red line). Generally accepted normal angles range from 120° to 145°. The critical angle of Gissane enlarges with greater posterior calcaneal facet collapse

Initially described in 1946 by Gissane [27], this angle measures depression of the posterior calcaneal facet. Proper measurement technique is performed on lateral foot radiographs, though lateral ankle radiographs can be used for screening purposes. The angle is formed by lines drawn along the superior surface of the anterior calcaneal process and along the posterior calcaneal facet (Fig. 8) [1]. In severely comminuted depressed fractures, this angle is not commonly utilized, as it can be difficult or impossible to visualize the bony landmarks, and measurements are considered unreliable [28].

The originally reported normal values are difficult to trace, and subsequent studies have reported a wide range of normal values from 95° to 150° [28]. In practice, generally accepted normal angles range from 120° to 145° [1]. The critical angle of Gissane increases with greater posterior calcaneal facet collapse.

#### Posterior tibialis tendon dysfunction

The posterior tibialis tendon serves as a major stabilizer in maintaining the medial longitudinal arch of the foot and resisting valgus forces on the hindfoot. Dysfunction of the posterior tibialis tendon, may start with tenosynovitis and tendinopathy without or with tendon tearing. Loss of stabilization by the posterior tibialis tendon places increased stress on the remaining medial longitudinal arch stabilizers, and failure of this tendon can result in progressive flatfoot deformity, forefoot abduction, acquired hindfoot valgus and varus talar tilt. Posterior tibialis tendon dysfunction is the most common cause of adult acquired flatfoot deformity [29]. Comprehensive discussion of the progression of adult acquired flatfoot deformity is beyond the scope of this article. However, it has been divided into 4 clinical stages. In stage I, there may be tenosynovitis and pain without deformity. In stage II, the posterior tibialis tendon begins to elongate, and there is mild hindfoot valgus. Stage II is often divided into stage IIA, in which there is mild flatfoot deformity, and the talar head uncoverage measures between 25 and 40%, and stage IIB, which represents a more severe flatfoot deformity, including hindfoot valgus measuring > 15° and > 40% talar head uncoverage. Stage III flatfoot deformity is rigid and there is often osteoarthritis (OA) in the hindfoot joints. Finally, stage IV disease represents stage III disease with valgus deformity at the ankle [30, 31]. Initial weightbearing radiographic and CT imaging focuses on detecting associated secondary alignment changes. MRI may be helpful to directly assess the status of the tendon and suggest associated malalignment. However, any malalignment detected on MRI should be confirmed on weightbearing studies.

**Hindfoot valgus and pes planus**

**Hindfoot valgus (tibiocalcaneal) angle**

**Fig. 9:**
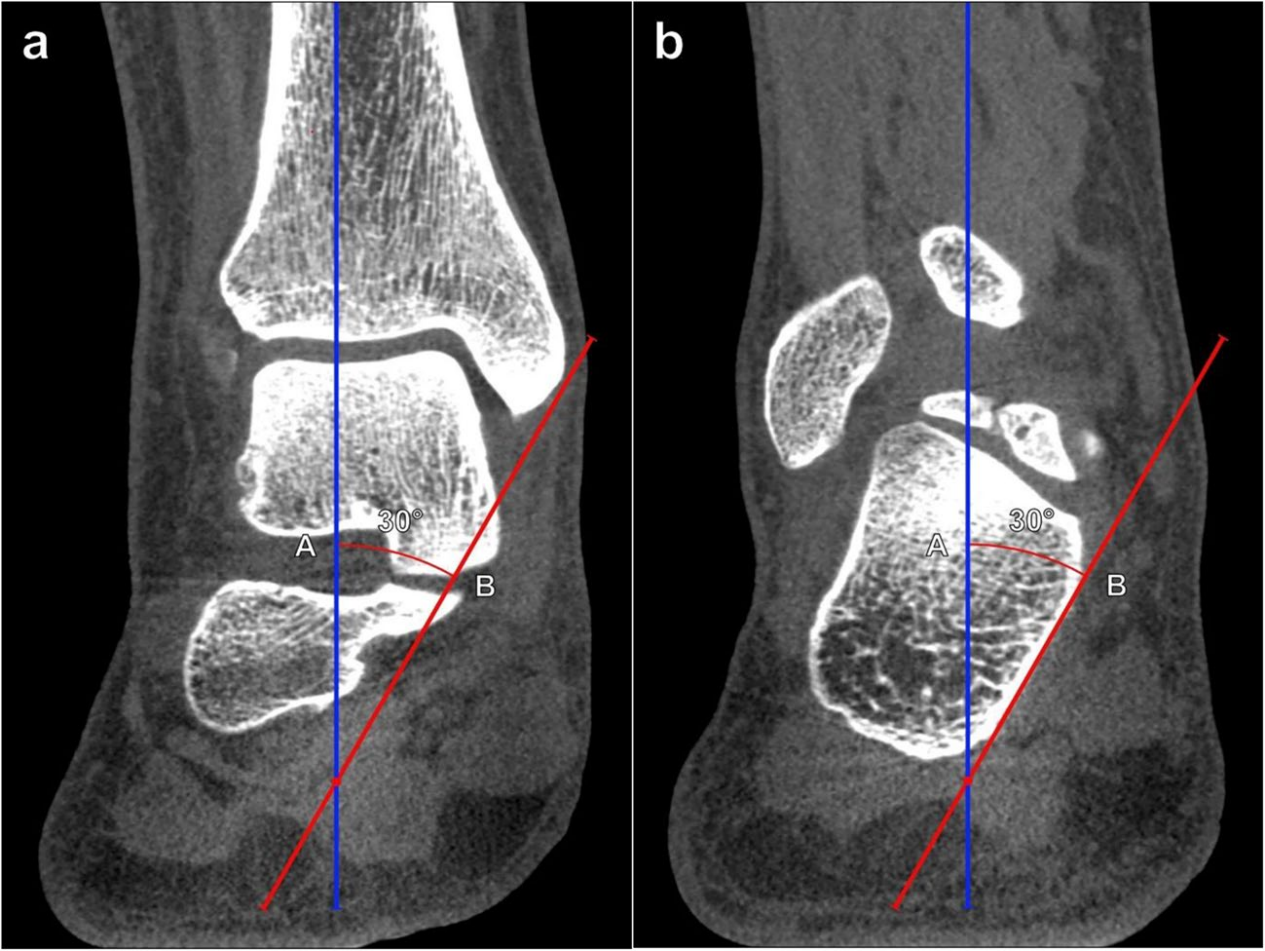
Tibiocalcaneal (hindfoot valgus) angle on coronal reformatted cone beam CT (CBCT) of the ankle: CBCT images are shown at the level of the distal tibia (**a**), which depicts the tibial axis (A, blue line) and at the level of the calcaneus (**b**), which shows the appropriate reference for the medial calcaneal cortex (B, red line). The angle is formed between lines drawn along the axis of the distal tibia (A) and tangent to the medial border of the calcaneus (B). Please note that the reference lines are drawn on two separate coronal reformatted images when using CBCT images and need to be superimposed to determine the angle. The hindfoot valgus angle normally measures ≤ 6° utilizing weightbearing radiography or CT, and larger angles indicate greater degrees of hindfoot valgus, which is associated with loss of the medial midfoot arch and posterior tibialis tendon dysfunction

Hindfoot alignment can be assessed on radiography or cross-sectional imaging by evaluating the angle between the tibial axis and the calcaneal axis. Weightbearing is important for obtaining reproducible, physiologically relevant measurements, because the calcaneus may either be in neutral or even varus angulation when in the relaxed, non-weightbearing state. Hindfoot valgus represents a lateral deviation of the calcaneal axis from the tibial midline, leading to forefoot abduction and talocalcaneal angle enlargement. Dedicated hindfoot alignment radiographs, initially described by Cobey et al. [32], can be utilized to minimize overlap of hindfoot structures by the forefoot. The hindfoot valgus (tibiocalcaneal) angle is measured between a line along the distal tibial longitudinal axis and a line tangential to the medial calcaneal cortex (Fig. 9). The hindfoot valgus angle normally measures ≤ 6° utilizing weightbearing radiography or CBCT [33]. An MRI grading system for the hindfoot valgus angle has been proposed, which classifies mild hindfoot valgus as 7°–16°, moderate as 17°–26°, and severe as > 26°, though it is important to remember that non-weightbearing MRI may underestimate the true degree of hindfoot angulation [34].

**Lateral talocalcaneal angle**

**Fig. 10:**
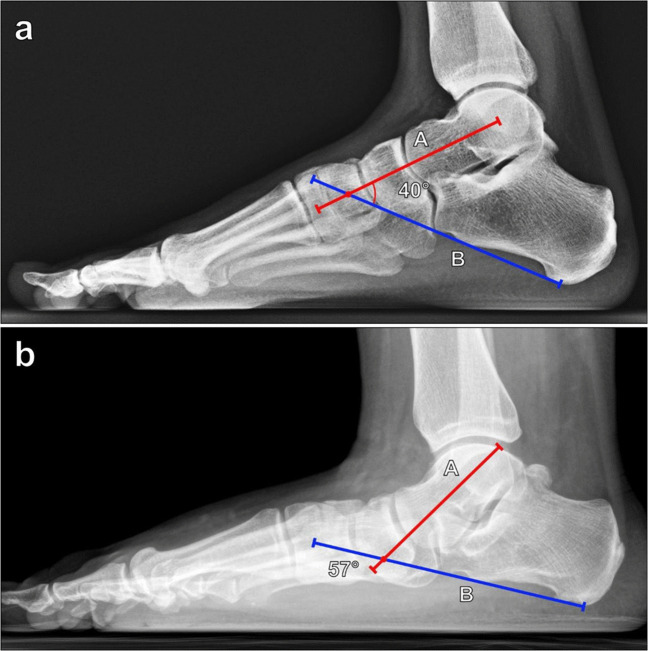
Measurement of the lateral talocalcaneal angle on lateral foot radiograph. **a** The angle is formed between a line (A, red line) along the axis of the talar neck and a line (B, blue line) along the inferior surface of the calcaneus. Normal values are between 25° and 40°. Values < 25° are seen in pes cavus, hindfoot varus, and congenital talipes equinovarus, while values > 40° are seen in pes planus and hindfoot valgus. **b** In a 34-year-old male patient with posterior tibialis tendon dysfunction and flatfoot deformity, the lateral talocalcaneal angle measures 57°, indicating pes planus and hindfoot valgus

The lateral talocalcaneal angle is used to assess hindfoot alignment and deformity. Measured on lateral weightbearing foot radiographs, it is defined as the angle between a line along the long axis of the talar neck and a line along the inferior surface of the calcaneus (Fig. 10). Normal values are between 25° and 40° with values < 25° seen in pes cavus, hindfoot varus, and congenital talipes equinovarus. Values > 40° are seen in pes planus and hindfoot valgus [1].

**Lateral talus-first metatarsal (Meary) angle**

**Fig. 11:**
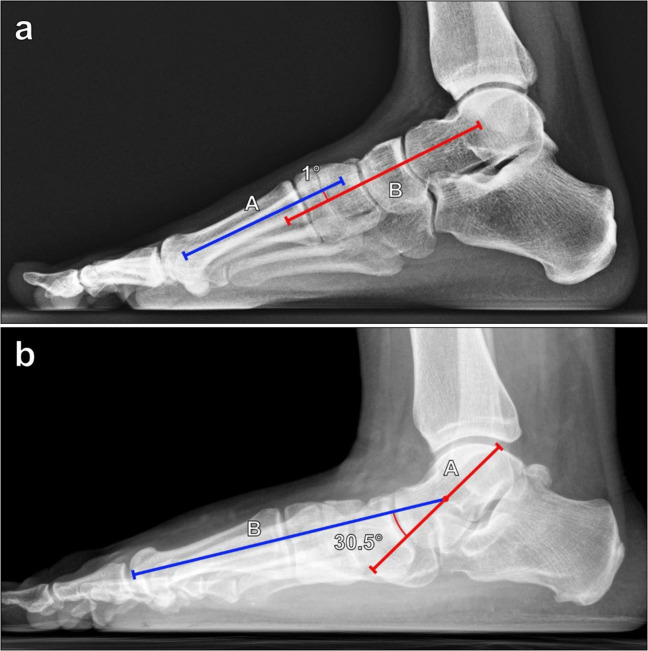
Measurement of the lateral talus-first metatarsal (Meary) angle on lateral weightbearing radiograph of the foot. **a** The angle is formed between lines drawn along the axis of the first metatarsal (A, blue line) and along the axis of the talus (B, red line). The long axis of the talus is either colinear or nearly colinear with the long axis of the first metatarsal, resulting in a normal angle range of 0° ± 4°. Angles greater than 4° convex downward are seen with pes planus, and angles greater than 4° convex upward are consistent with pes cavus. **b** In a 34-year-old male patient with posterior tibialis tendon dysfunction and flatfoot deformity (same patient as in Fig. 10b), the lateral talus-first metatarsal angle measures more than 30°, indicating borderline severe pes planus

The lateral talus-first metatarsal angle, initially described by Meary et al., is measured on a lateral weightbearing foot radiograph since non-weightbearing radiographs may underestimate the degree of deformity. The angle is formed by a line along the long axis of the talar head and neck and a line along the long axis of the first metatarsal (Fig. 11). Normally, the long axis of the talus is colinear with the long axis of the first metatarsal, resulting in a normal angle range of 0° ± 4° [35]. Angles greater than 4° convex downward are consistent with pes planus, and angles greater than 4° convex upward are consistent with pes cavus. Pes planus can be graded as follows: 5°–15° is mild; 16°–30° is moderate; and > 30° is severe [36].

**Calcaneal inclination (pitch) angle**

**Fig. 12:**
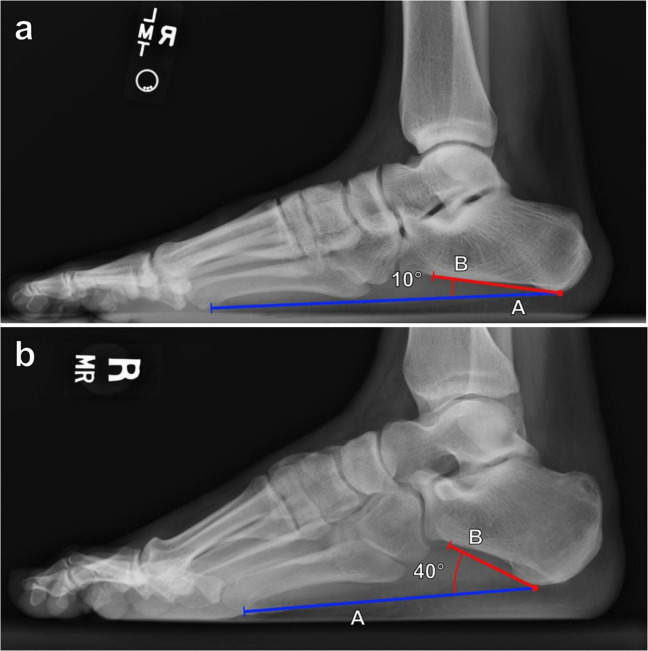
Measurement of the calcaneal inclination (pitch) angle on lateral weightbearing radiograph of the foot. The angle is formed between a horizontal line (A, blue line) from the calcaneal tuberosity to the plantar surface of the 5th metatarsal head, and a line (B, red line) along the inferior surface of the calcaneus. Reported normal values are variable although 20°–30° is often considered normal. A calcaneal inclination angle measuring < 20° is consistent with pes planus, while an angle > 30° suggests of pes cavus. **a** Pes planus in a 24-year-old male patient with a 10° calcaneal inclination angle. **b** Pes cavus in a 30-year-old female patient with a 40° calcaneal inclination angle

The calcaneal inclination (pitch) angle is used to assess arch height. Measured on lateral weightbearing foot radiographs, it is defined as the angle formed from a line along the plantar surface of the calcaneus and a line from the plantar margin of the calcaneal tuberosity to the plantar surface of the 5th metatarsal head, representing the plantar weightbearing axis of the foot (Fig. 12). Non-weightbearing lateral radiographs may underestimate the degree of the deformity. There has been significant variability in the normal values reported, with values of 20°–30° often considered normal [37, 38]. A decreased calcaneal inclination angle (< 20°) is consistent with pes planus, while an increased angle (> 30°) suggests of pes cavus.

**Forefoot abduction**

**Talonavicular uncoverage percentage**

**Fig. 13:**
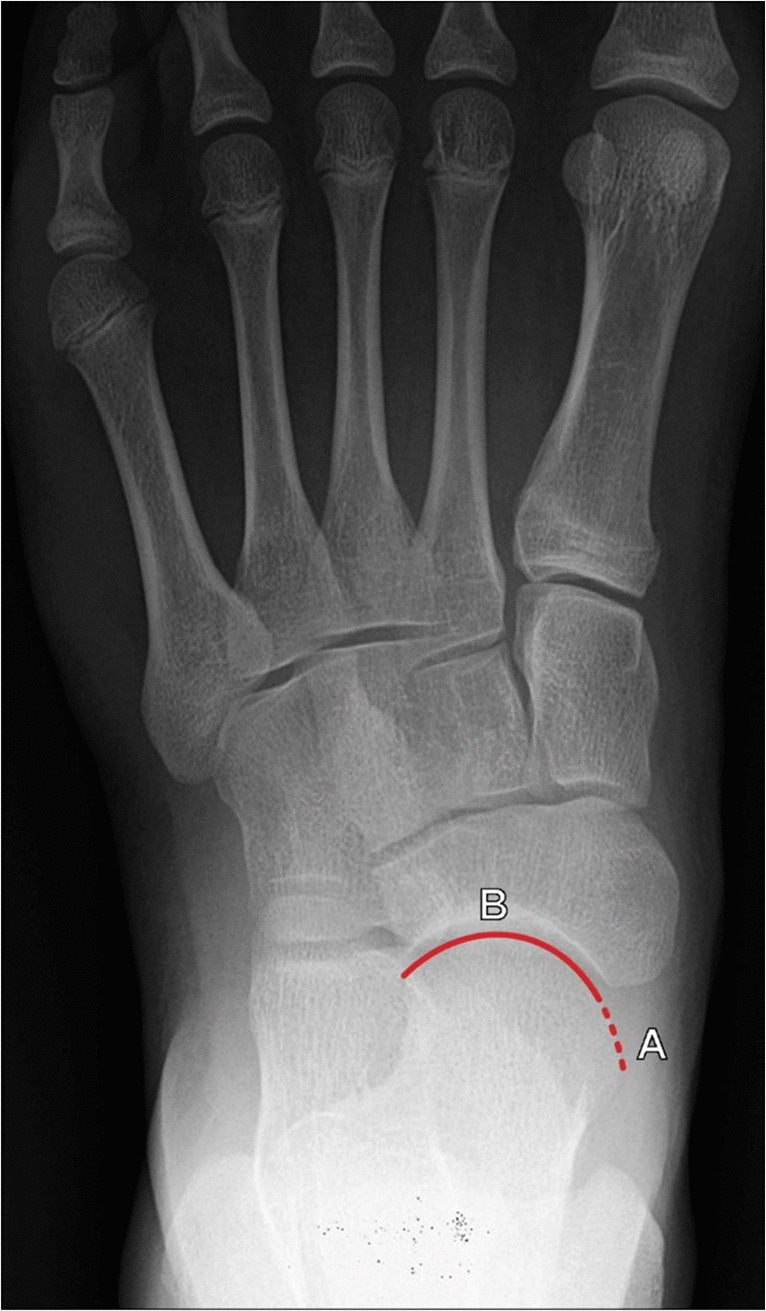
Measurement of the Talonavicular Uncoverage Percentage on DP radiograph of the foot. The portion of the talar head articular surface that is not covered by the navicular (A, dotted line) is divided by the length of the total talar head articular surface (A + B) and multiplied by 100% to give a percentage. A Talonavicular Uncoverage Percentage > 30% is consistent with pathologic flatfoot deformity, and is used to differentiate between stage IIA and stage IIB acquired flatfoot deformity

The talonavicular uncoverage percentage represents the portion of the medial talar head articular surface that is uncovered by the navicular articular surface at the talonavicular joint. This measurement is performed on a dorsoplantar (DP) weightbearing radiograph of the foot (Fig. 13) because non-weightbearing radiographs may underestimate the degree of uncoverage. The talonavicular uncoverage percentage is determined by dividing the length of the talar head articular surface that is uncovered by the navicular by the total length of the talar articular surface, which is then multiplied by 100% to give a percentage. A talonavicular uncoverage ercentage > 30% is considered consistent with pathologic flatfoot deformity, and this cutoff is used as the differentiator between stage IIA and stage IIB acquired flatfoot deformity, which can have implications for surgical management [39].

**Less commonly used measurements to assess hindfoot alignment and forefoot abduction**

**AP talocalcaneal angle**

**Fig. 14:**
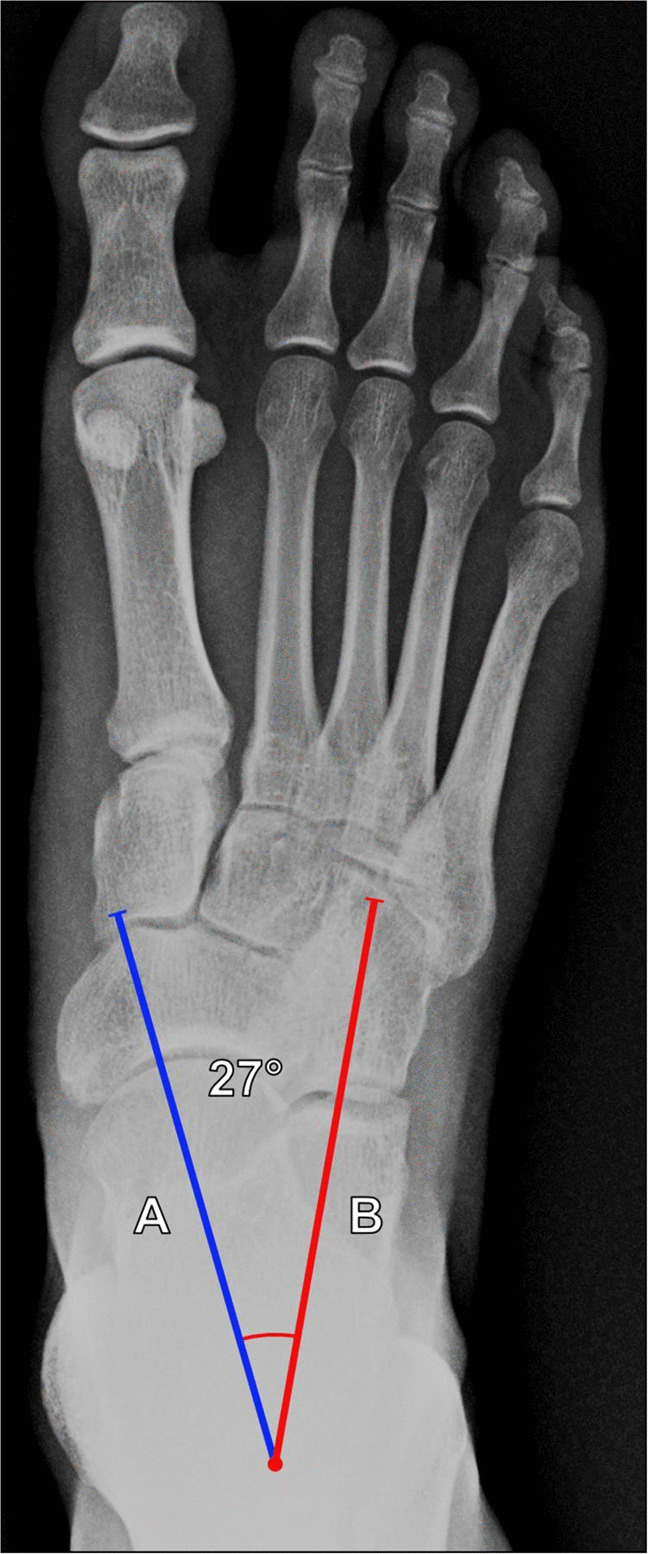
Measurement of the AP talocalcaneal (Kite) angle on DP weightbearing radiograph of the foot. The angle is formed between a line (A, blue line) along the talar axis and a line (B, red line) along the calcaneal axis. Normal values range between 25° and 40°. Angles < 25° suggest hindfoot varus, and those > 40° suggest hindfoot valgus

Also known as the Kite angle, this is commonly used to evaluate hindfoot deformity and can be useful in assessing hindfoot varus and valgus, as well as to evaluate for successful correction of clubfoot deformity. The AP talocalcaneal angle is measured on DP weightbearing foot radiographs. The angle is formed from lines drawn along the talar and calcaneal long axes. Normal values range between 25° and 40°. Angles < 25° suggest hindfoot varus, and those > 40° suggest hindfoot valgus (Fig. 14) [38, 40].

**Talonavicular coverage angle**

**Fig. 15:**
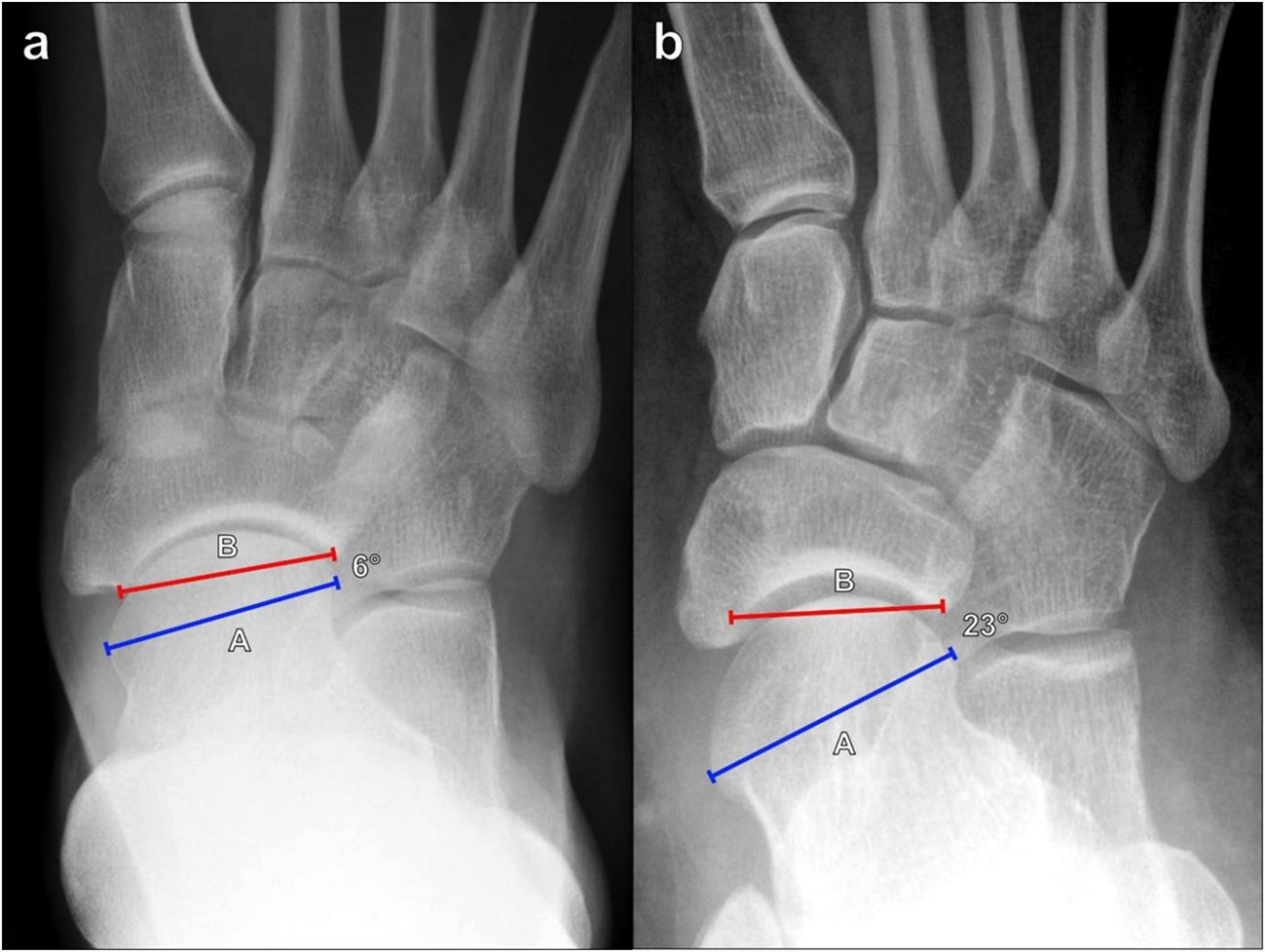
Measurement of the talonavicular coverage angle on DP weightbearing radiograph of the foot. **a** The angle is taken between a line joining the medial and lateral margins of the talar head articular surface (A, blue line) and a line joining the medial and lateral margins of the navicular base articular surface (B, red line). Values between 2 and 20° are usually considered normal, while values > 20° are associated with abnormal forefoot abduction and pes planus. There is good correlation of this measurement on both weightbearing radiographs and axial weightbearing cone beam CT (CBCT) images; measurements made on axial CBCT images should be performed at the level where the talonavicular joint is fully included in the field-of-view. **b** In a 34-year-old male patient with posterior tibialis tendon dysfunction and flatfoot deformity (same patient from Figs. 10b and 11b), the talonavicular coverage angle measures 23°, indicating forefoot abduction and pes planus [1, 41–43].

This angle can be utilized to evaluate excessive forefoot abduction by assessing the angle between the talar and the navicular articular surfaces. Measurement of the talonavicular coverage angle is performed on DP weightbearing foot radiographs (Fig. 15). The angle is taken from a line joining the medial and lateral margins of the talar head articular surface and a second line joining the medial and lateral margins of the navicular base articular surface. Although there is variation in reported cutoffs, values between 2° and 20° are generally considered normal, and values > 20° are associated with abnormal forefoot abduction and flatfoot. This measurement has shown good correlation with weightbearing radiographs when measured on axial weightbearing CBCT images at the level where the talonavicular joint is fully included in the field of view [1, 41–43].

**Talonavicular incongruency angle**

**Fig. 16:**
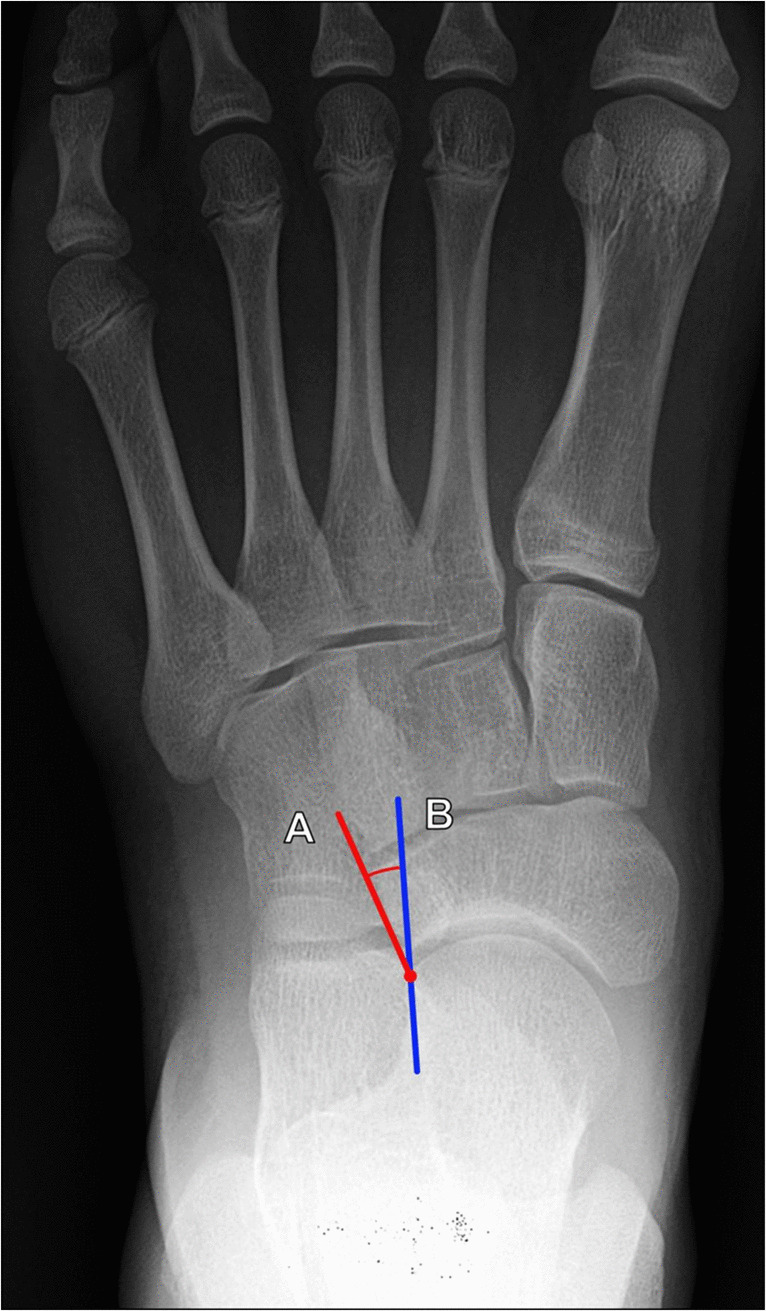
Measurement of talonavicular incongruency angle on DP weightbearing radiograph of the foot. The angle is formed from a line connecting the lateral aspects of the talar and navicular articular surfaces (A, red line) and a line connecting the lateral margin of the talar neck at its narrowest segment and the lateral talar articular surface (B, blue line). A larger incongruency angle indicates greater forefoot abduction and more severe pes planus, and a measured value > 45° reflects stage IIB flatfoot deformity

The talonavicular incongruency angle is a newer measurement for evaluating forefoot abduction. This measurement is performed on DP weightbearing radiographs of the foot and formed between a line connecting the lateral aspects of the talar and navicular articular surfaces and a line connecting the lateral margin of the talar neck at its narrowest segment and the lateral talar articular surface (Fig. 16). An initial study of this angle by Ellis et al. reported a range of normal values fell between −5° and 20°, and values > 45° were considered representative of the IIB stage of flatfoot deformity. This study indicated that the talonavicular incongruency angle with a cutoff of > 50° demonstrates the most significant difference for patients with stage IIB disease [41, 44].

**AP talo-first metatarsal angle**

**Fig. 17:**
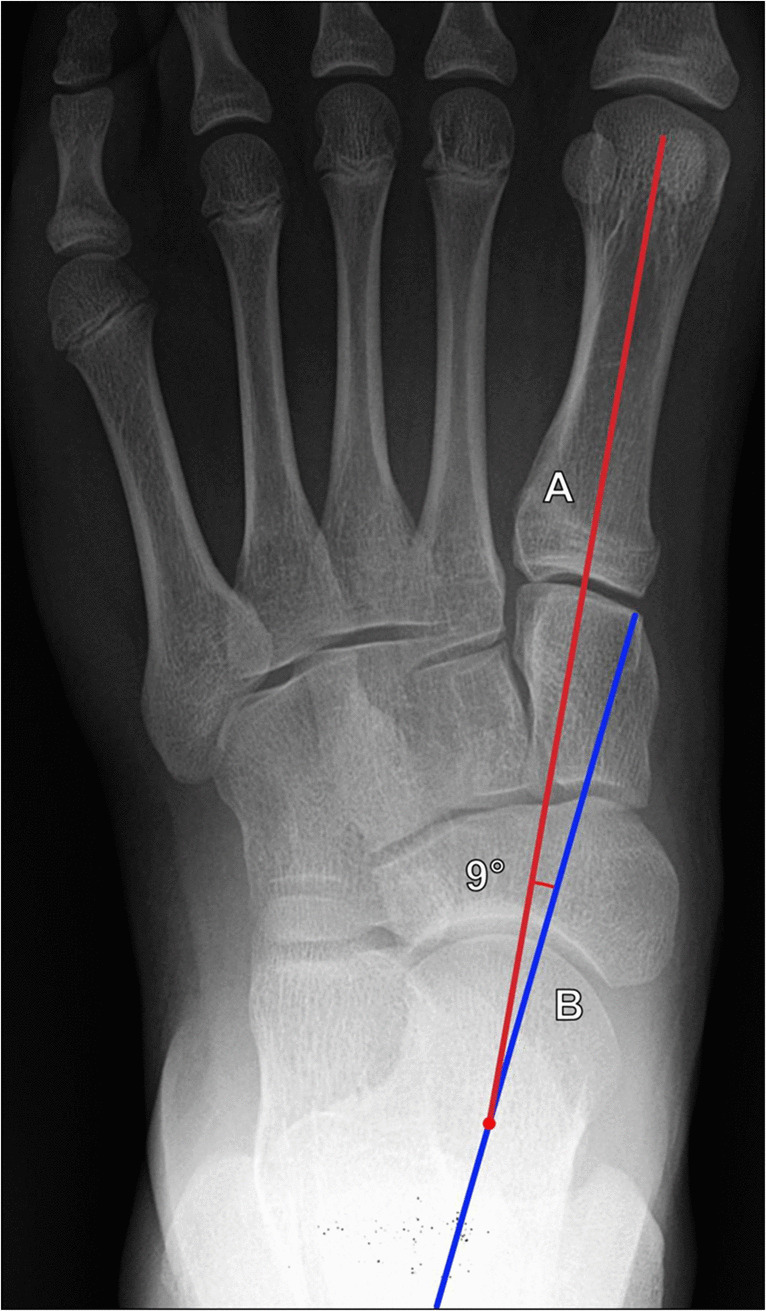
Measurement of AP talo-first metatarsal angle on DP weightbearing radiograph of the foot. The angle is measured between a line (A, red line) extending along the long axis of the first metatarsal and a line (B, blue line) along the long axis of the talar neck. Normal values are approximately 7° ± 4°, and higher values are associated with excessive forefoot abduction

As shown in Fig. 17, this measurement evaluates forefoot abduction by assessing the angle between the long axes of the talus and the first metatarsal. Measurement of AP talo-first metatarsal angle is performed on DP weightbearing radiographs of the foot, and the angle is measured from a line extending along the axis of the first metatarsal and a line along the axis of the talar neck. Normal values are approximately 7° ± 4°, with higher values associated with excessive forefoot abduction especially in the setting of forefoot deformity. This measurement can also be made on weightbearing axial CBCT images [38, 43].

#### Arthroplasty

**Fig. 18:**
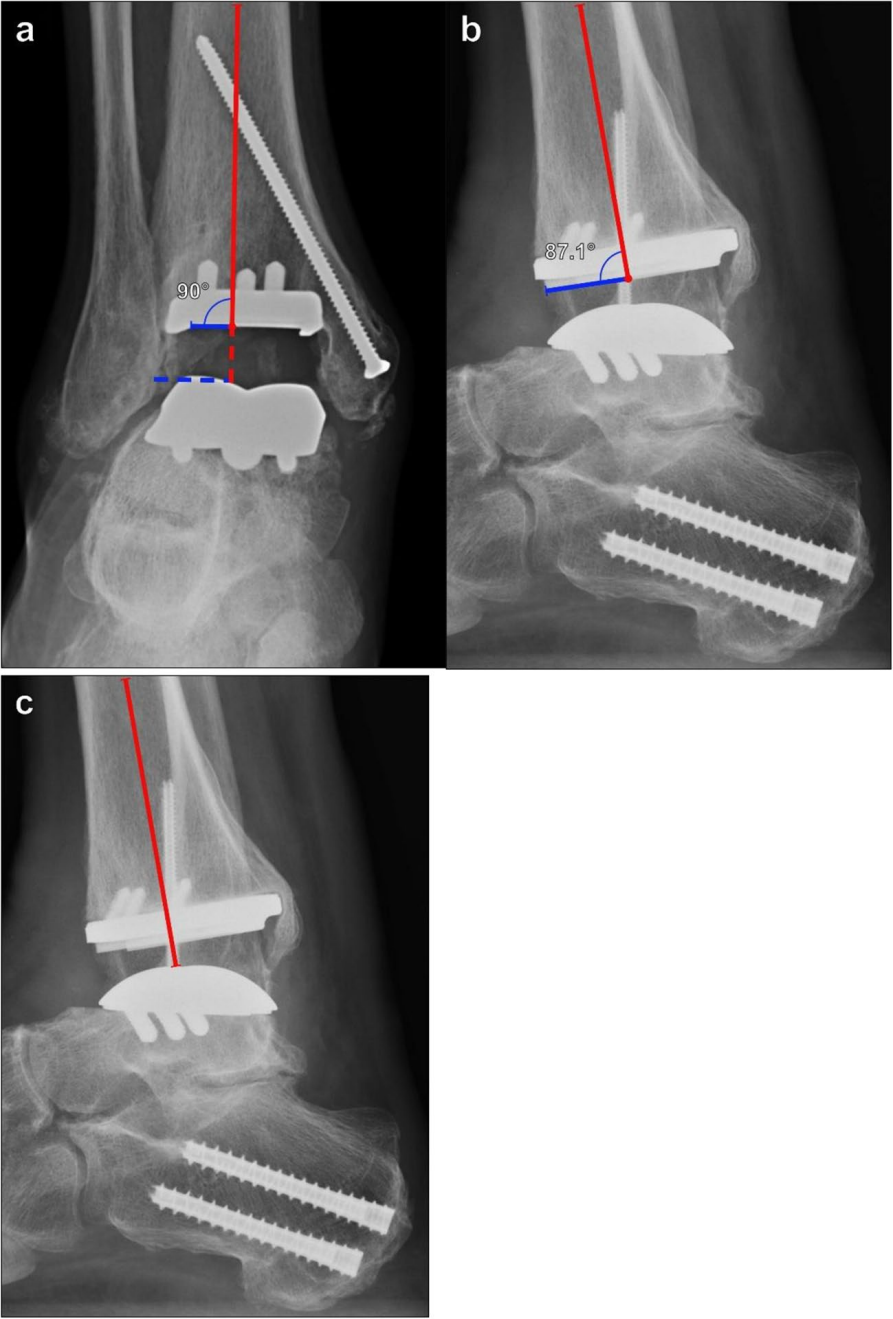
Example of postoperative radiographic appearance of ankle arthroplasty. **a** On AP radiograph, the articular surface of the tibial component (solid blue line) and axis of the talar component surface (dashed blue line) should be 90° relative to the long axis of the tibia (solid red line). **b** On lateral radiograph, the articular surface of the tibial plafond component (blue line) should be 89 ± 3° relative to the long axis of the tibia (solid red line). **c** On lateral view, approximately 40–45% of the talar component should be anterior to the mid-tibial line along the tibial axis (red line). These parameters help to identify device malalignment or early component migration

There are more than 20 total ankle arthroplasty (TAA) designs, which largely precludes standardization of post-operative measurement [45, 46]. Prosthetic migration can be difficult to perceive on individual images, and serial AP and lateral weightbearing ankle radiographs with standardized technique are critical to producing reproducible measurements that may only be discernable over successive studies. Because of the complex anatomy of the hindfoot, weightbearing CBCT with metal artifact reduction techniques is useful to detect areas of periprosthetic osteolysis or confirm prosthetic migration/rotation when radiographs are inconclusive. In general, on AP radiographs (Fig. 18a) the tibial component articular surface should be 90° to the anatomic axis of the tibia. The talar component should also be 90° relative to the anatomic axis of the tibia, with a change of ≥ 10° either medially or laterally considered pathologic [47].

On lateral radiographs (Fig. 18b-c), the AP distal tibial angle is measured by the angle formed by the anatomic axis of the tibia and a line along the articular surface of the tibial component and should be 89° ± 3° [48]. The tibial prosthesis should cover the cut plafond surface in the AP dimension [49]. On lateral weightbearing views, 40–45% of the talar component should be anterior to a vertical line through the long axis of the tibial diaphysis [45].

Aseptic loosening and periprosthetic osteolysis are the most common long-term complications of TAA, and post-arthroplasty imaging should assess for these complications. Radiographically, these complications manifest as periprosthetic lucency; > 2 mm of periprosthetic lucency raises concern for aseptic loosening or periprosthetic osteolysis and should be followed closely for progression [45]. Subsidence, or migration of the prosthesis related to bone collapse in the axis of loading force, more commonly involves the talar component and is defined as a change in the vertical position of the tibial or talar component by ≥ 5 mm [50].

## Forefoot

### Imaging modalities

#### Radiography

The DP weightbearing foot radiograph is the most commonly used projection for measurements of the foot. The foot should be pointed straight forward in neutral rotation with the beam centered on the midfoot at the tarsometatarsal articulations [51]. The X-ray beam can be angled 10°–15° posteriorly toward the heel to open the tarsometatarsal joints when there is concern for midfoot OA or Lisfranc trauma [52]. Standing lateral radiographs are taken with evenly distributed weight between both feet and the X-ray beam focused on the level of the navicular tuberosity [53]. The medial and lateral superior talar dome should overlap, and the metatarsals should nearly overlap with the exception of the base of the fifth metatarsal. Consistent foot orientation and true weight distribution are critical in obtaining reproducible, standardized measurements, and improper patient position, leading to X-ray beam obliquity, forefoot pronation/supination or abduction/adduction, or lack of physiologic weightbearing for a particular patient can distort the anatomy, precluding appropriate measurements.

#### Cross-sectional imaging

Cross-sectional imaging is used similarly when evaluating the foot as with the ankle. CT provides optimal osseous detail with the capability for multiplanar and 3-dimensional volumetric reformats, and is often used for fracture evaluation, pre-operative planning, and post-operative evaluation. As described previously, conventional MDCT is performed without weightbearing, which limits its utility and accuracy in evaluating the many forefoot pathologies that are exacerbated by weightbearing. Weightbearing CBCT of the foot is becoming more commonly clinically available, since it images the foot in a more physiologic standing position and improves measurement accuracy, such as for assessment of hallux valgus and hallux sesamoid rotation [4, 5]. Additionally, CBCT images are often acquired with thin sections, which can allow reconstruction in any imaging plane at the workstation. As a result, CBCT may be less reliant than radiography on proper patient positioning. Because of these factors, weightbearing CBCT has become an important tool for foot and ankle surgeons in preoperative planning and postoperative serial assessment.

MRI is primarily used for evaluation of the soft tissue structures of the foot; as this examination is performed non-weightbearing, the ability to make accurate osteoarticular measurements is limited. Similar to the ankle and hindfoot, although some radiographic measurements can be applied to MRI to suggest diagnoses, the results should be confirmed on weightbearing radiography or CBCT.

### Measurements (Table 2)

**Table 2:** Commonly used orthopaedic measurements in assessment of the foot

Radiologic measurement	Definition	Imaging modality of choice	Normal value(s)/normal range	Abnormal value(s)/clinical significance
Sgarlato’s angle/metatarsus adductus angle	Measure of metatarsus adductus, or medial deviation of the forefoot relative to the hindfoot (Fig. 20)	DP foot XR	0°–15°	**·**15°–19°-mild metatarsus adductus **·**20°–25°- moderate metatarsus adductus **·** > 25°severe metatarsus adductus
Hallux valgus angle (first metatarsophalangeal angle)	Angle formed by lines along the anatomic axes of the first metatarsal and first proximal phalanx (Fig. 21)	DP foot or great toe XR	< 15°	**·**15°–20° mild hallux valgus **·**21°–39° moderate hallux valgus **·** ≥ 40° severe hallux valgus
Hallux valgus interphalangeus angle	Angle formed by lines along the anatomic axes of the first distal and proximal phalanges (Fig. 22)	DP foot or great toe XR	< 10°	≥ 10° hallux valgus interphalangeus
First–second intermetatarsal angle	Angle formed by lines along the anatomic axes of the first and second metatarsals (Fig. 23)	DP foot XR	< 9°	**·**9°–11° mild hallux valgus **·**12°–17° moderate hallux valgus **·** ≥ 18° severe hallux valgus
Distal metatarsal articular angle (DMAA)	Angle formed by a line perpendicular to a line connecting the first metatarsal head medial and lateral articular surfaces and a line along the first metatarsal axis (Fig. 24)	DP foot XR	≤ 10°	> 10° has been correlated with progressive hallux valgus
Sesamoid rotation angle	Angle formed by a line connecting the most inferior aspect of the medial and lateral sesamoids (XR) or along the widest portion of the sesamoid apparatus (CT/MR), and a line along the weight bearing surface of the forefoot (Fig. 25)	AP sesamoid tangential XR or Coronal CBCT reformatted image	0°–13°	> 13° excessive lateral sesamoid rotation, associated with greater hallux valgus
First metatarsal pronation angle	Angle formed by a line drawn from the lateral most edge of the lateral sesamoid sulcus to the medial-most edge of the medial sesamoid sulcus, and a line along the weight bearing surface of the forefoot (Fig. 26)	AP sesamoid tangential XR or Coronal CBCT reformatted image	−4°–12°	**·** > 12° greater metatarsal head pronation **·** < −4° greater metatarsal supination
Metatarsophalangeal joint angle	Angle formed by lines connecting the medial and lateral margins of 1st metatarsal head articular surface and medial and lateral margins of proximal phalanx base articular surfaces (Fig. 27)	DP foot or great toe radiographs	< 10°	≥ 10° indicates joint incongruence
Congruence index	Percentage of the first metatarsal head that is overlapped by the first proximal phalanx base (Fig. 28)	DP foot or great toe radiographs	> 76.5%	≤ 76.5% indicates joint incongruence

#### Lisfranc alignment

The Lisfranc joint complex provides stability to the midfoot, and injury to this skeletal and ligamentous complex can lead to long term structural malalignment and degenerative arthropathy of the midfoot. Radiographic findings of Lisfranc joint complex injury can be very subtle; thus, careful evaluation of Lisfranc alignment is necessary. Occasionally, evidence of malalignment is only present on weightbearing views of the foot.

**Fig. 19:**
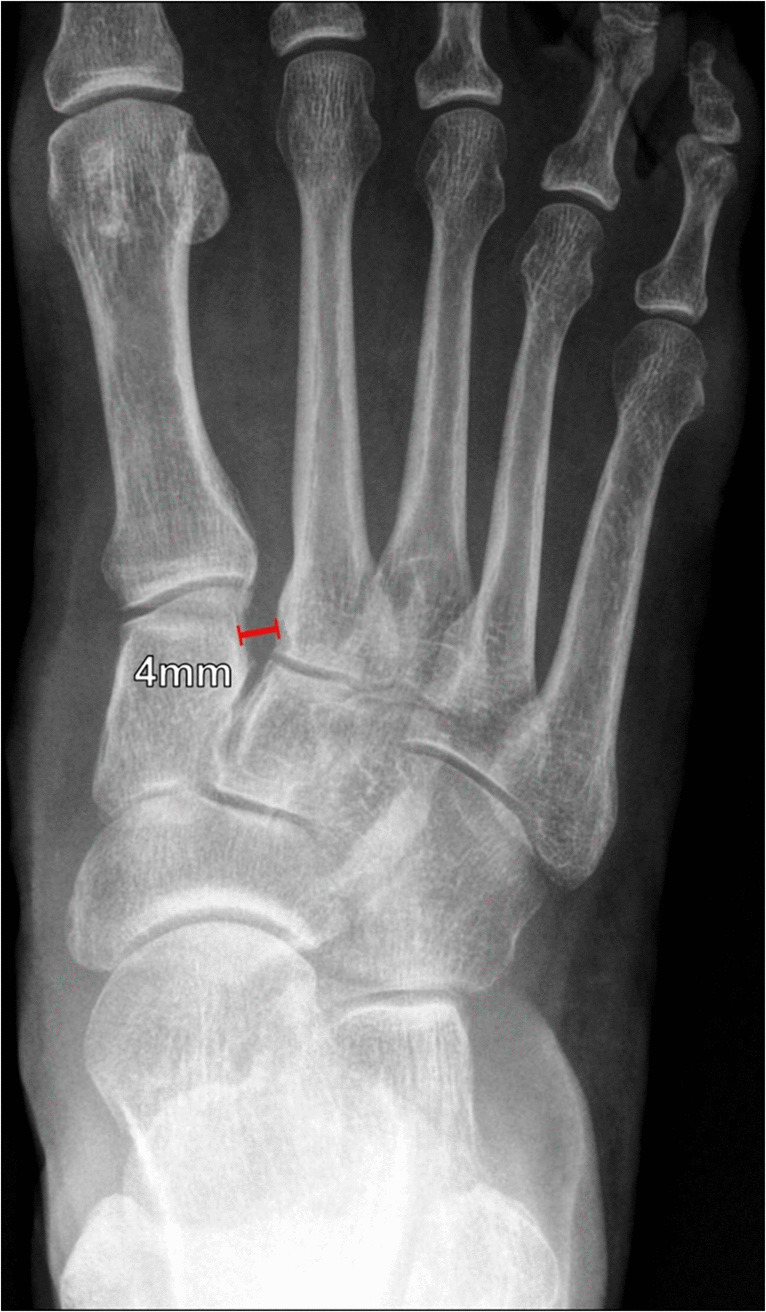
Lisfranc diastasis on DP weightbearing radiograph of the foot in a 21-year-old male football player following injury. Normally, the lateral margins of the medial cuneiform and the first metatarsal base are aligned, as are the medial margins of the middle cuneiform and second metatarsal base. The distance between the medial cuneiform and the second metatarsal base should measure ≤ 2 mm. A measurement > 2 mm or asymmetry compared with the asymptomatic contralateral foot suggests Lisfranc ligament insufficiency. In the imaging example, the space between the medial cuneiform and second metatarsal base measures more than 4 mm, indicating Lisfranc ligament complex insufficiency

On DP radiographs of the foot, when the Lisfranc joint complex is intact, the lateral margins of the medial cuneiform and the first metatarsal base should be in line with one another, as should the medial margins of the middle cuneiform and the second metatarsal base (Fig. 19). The distance between the medial cuneiform and the second metatarsal base should measure ≤ 2 mm; higher measurements suggest Lisfranc ligament insufficiency [52, 54]. In subtle cases, comparison with a DP radiograph of the contralateral foot can be helpful, as asymmetry with the contralateral foot also suggests Lisfranc ligament injury. Projectional rotation or partial weightbearing may obscure subtle diastasis or associated fractures.

Additionally, CT or MRI may help confirm ligamentous insufficiency and subtle, associated fractures when injuries are radiographically occult. Weightbearing CBCT can help detect osseous malalignment that may not be visible when the patient is supine. As with radiography, some patients require imaging both feet to detect subtle diastasis. MRI has the added benefit of directly visualizing the Lisfranc ligamentous complex and can help detect osteoarticular injuries, such as distal cuboid fractures, which are often associated with these injuries.

#### Metatarsus adductus

Medial deviation of the forefoot relative to the hindfoot results in metatarsus adductus. Though commonly seen in conjunction with other malalignment as part of congenital clubfoot deformities of infancy, it can occur in isolation in adults. The deformity is usually mild, flexible, and self-correcting; however, severe deformities may require treatment [55].

**Fig. 20:**
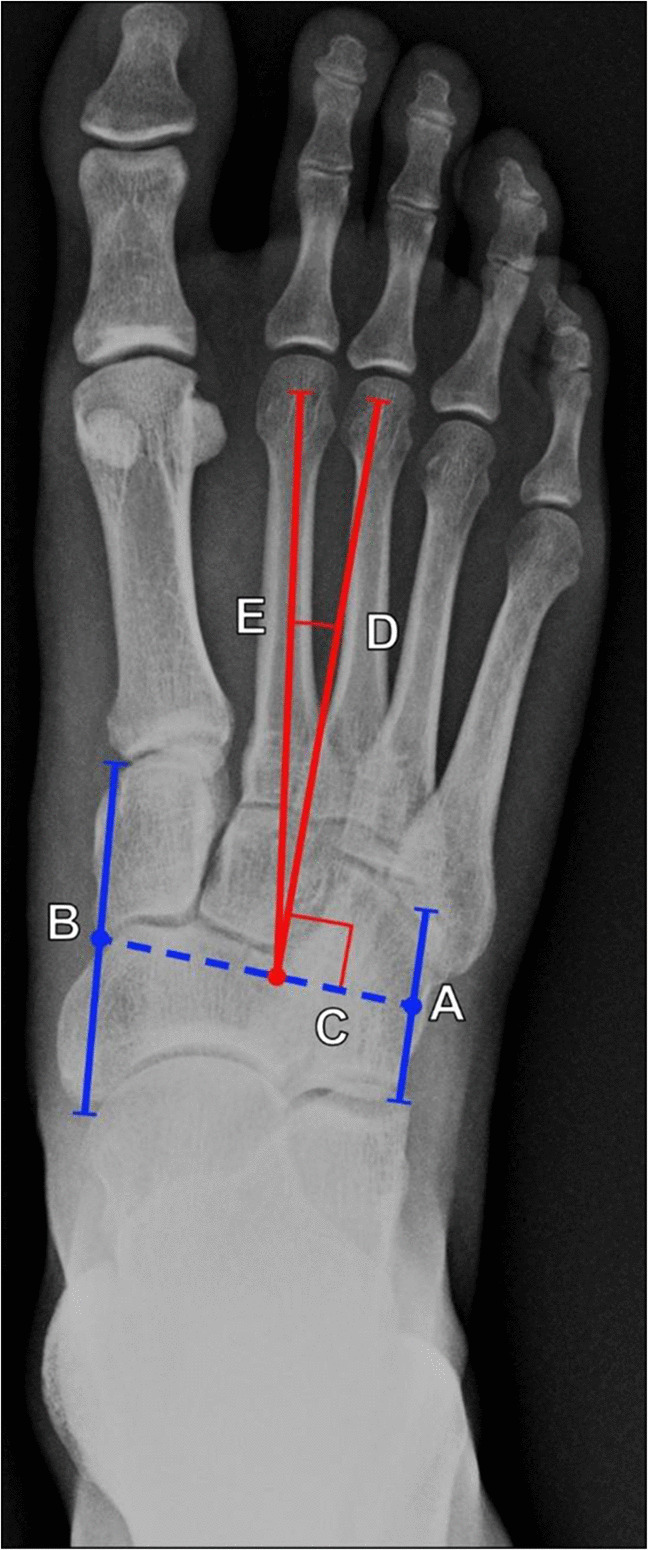
Measurement of metatarsus adductus (Sgarlato’s) angle on DP radiograph of the foot using the Sgarlato method. This is performed by drawing 5 lines on DP radiograph of the foot: a line (A, shorter solid blue line) connecting the lateral-most points of the calcaneocuboid and the fourth metatarso-cuboid joint, a line (B, longer solid blue line) connecting the medial most points of the talonavicular and medial cuneiform-first metatarsal joints, a line (C, dashed blue line) connecting the midpoints of line A and line B, a line (D, lateral red line) perpendicular to line C, and a line (E, medial red line) along the long axis of the second metatarsal. Sgarlato’s angle is formed between lines D and E. The normal Sgarlato’s angle measures 0°–15°, considered a "rectus" foot, while a foot with an angle > 15° is an "adductus" foot. The Sgarlato method has the highest inter- and intra-observer reliability when assessing metatarsus adductus

There is an array of methods to assess metatarsus adductus. The traditional Sgarlato method has the highest inter- and intra-observer reliability. It is measured on DP foot radiographs, and its method of measurement is depicted on Fig. 20 [56, 57]. Sgarlato’s angle is also known as the metatarsus adductus angle (MAA).

The normal Sgarlato’s angle measures 0°–15° [58]. A foot with a normal angle is considered a “rectus” foot while a foot with an angle > 15° is an “adductus” foot. Mild metatarsus adductus is 15°–19°, moderate metatarsus adductus is 20°–25°, and > 25° is severe metatarsus adductus. Medial deviation of the first metatarsal increases as the angle increases. Sgarlato’s angle is important for preoperative planning for hallux valgus correction since the larger this angle is, the more likely the second metatarsal is to block appropriate surgical correction of the first metatarsal, and more complex surgical corrections are needed.

#### Hallux valgus

Hallux valgus is a complex deformity involving the first ray, and a common cause of forefoot pain. Originally described in 1871 [59], it is the most common pathologic entity affecting the great toe [60]. It involves a varus deformity of the first metatarsal, a valgus deformity of the first metatarsophalangeal (MTP) joint, and pronation of the first toe [61]. There are several radiological measurements, including the hallux valgus angle, intermetatarsal angle and distal metatarsal articular angle, to diagnose and quantify the degree of deformity, and guide operative planning and postoperative follow-up. Weightbearing radiography is the imaging modality of choice, and proper technique and patient positioning are important to allow detection and grading of hallux valgus utilizing standardized reference ranges as well as help with preoperative planning.

##### Hallux valgus angle

**Fig. 21:**
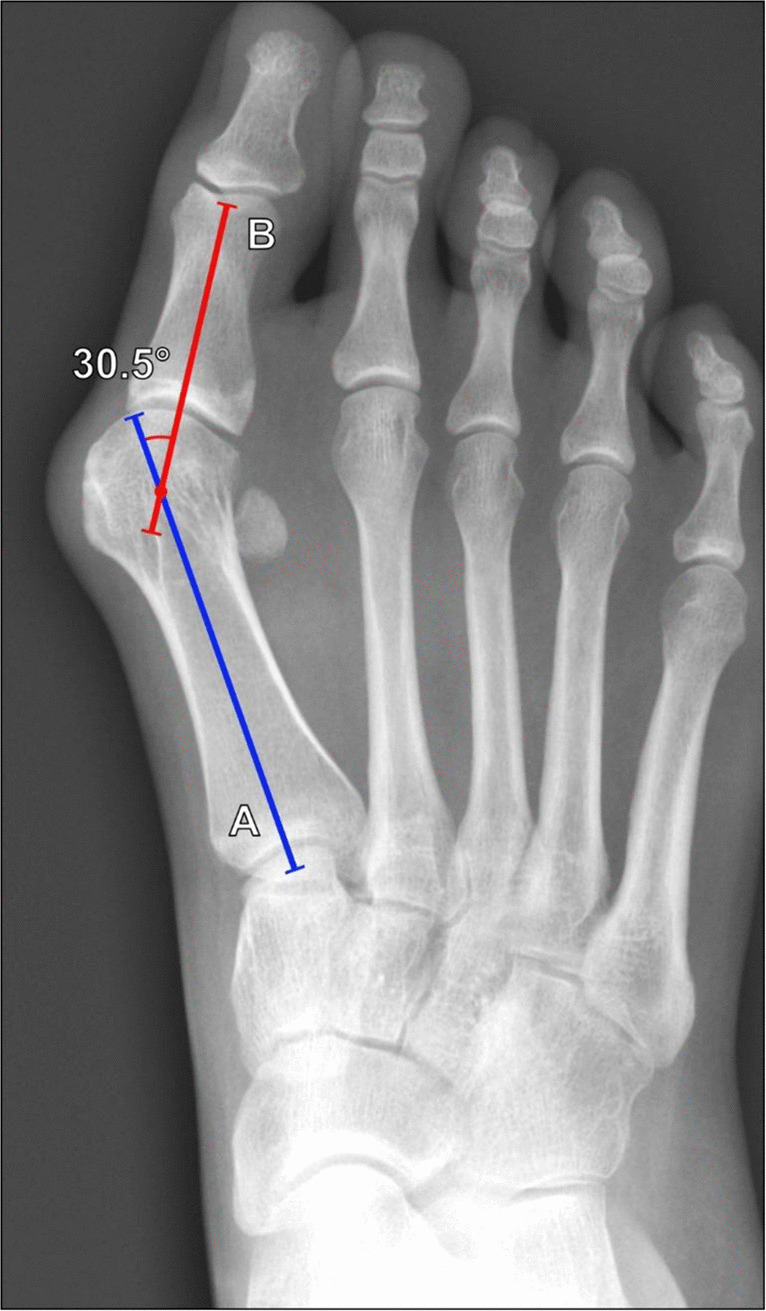
Measurement of hallux valgus (first metatarsophalangeal) angle on DP radiograph of the foot in a 32-year-old female patient with forefoot pain and deformity. The angle is formed between the long axis of the first metatarsal (A, blue line) and the first proximal phalanx (B, red line). The normal angle measures < 15°, and an angle ≥ 15° reflects hallux valgus. Radiographically, there is an increased first metatarsophalangeal angle, indicating moderate hallux valgus, with bunion formation and lateral hallux sesamoid rotation

The hallux valgus angle, also known as the first metatarsophalangeal angle, is the most common measurement made in assessing hallux valgus. This measures the amount of valgus deformity at the first MTP joint. Measured on DP weightbearing foot or great toe radiographs, the angle is formed by lines along the long axes of the first metatarsal and first proximal phalanx (Fig. 21). The longitudinal axes should be centered on perpendicular transverse reference lines located 1 cm from the articular surfaces of the metatarsal, and 0.5 cm from the articular surfaces of the proximal phalanx [51]. The angle normally measures < 15°; mild hallux valgus is 15°–20°; moderate hallux valgus is 21°–39°; and severe hallux valgus is ≥ 40° [62]. There is a strong correlation between measurements made on radiography and on weightbearing CBCT [63, 64].

##### Hallux valgus interphalangeus angle

**Fig. 22:**
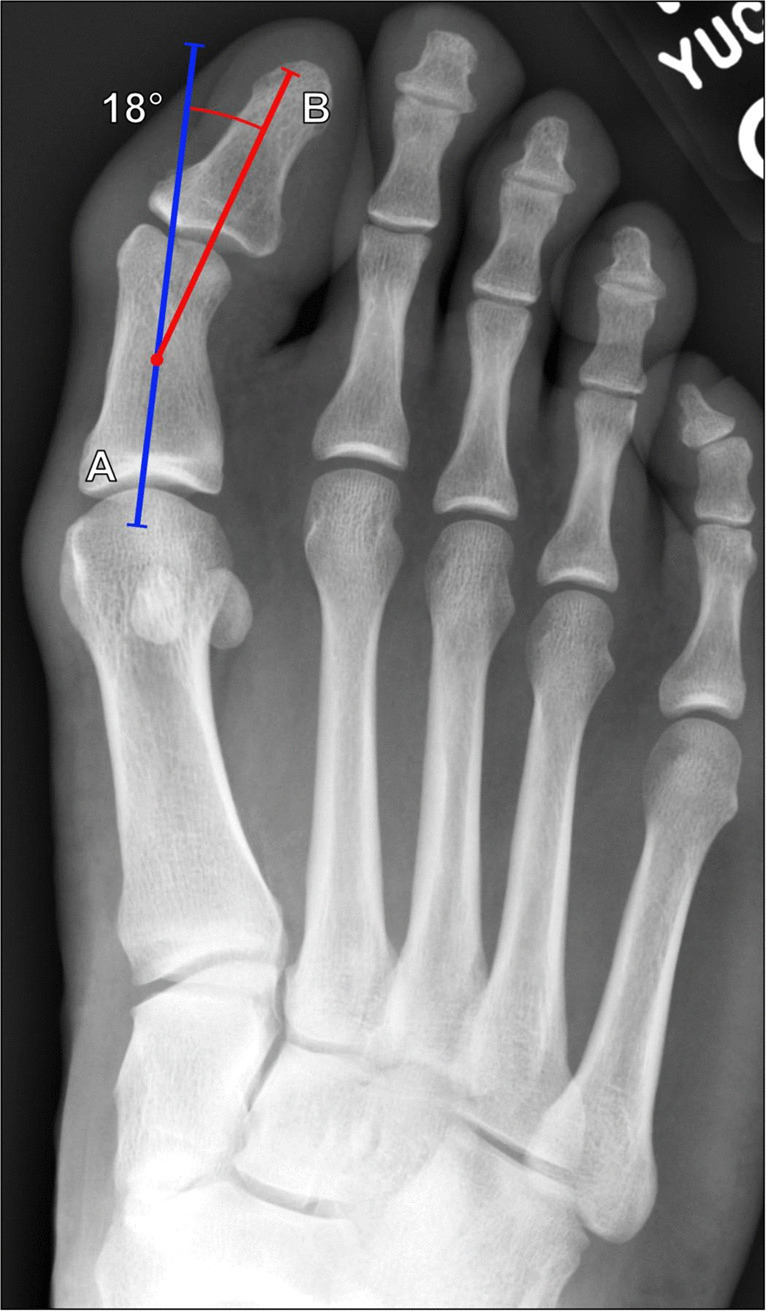
Measurement of hallux valgus interphalangeus angle on DP radiograph of the foot in an 18-year-old male basketball player with forefoot injury. The angle is formed between the long axis of the first proximal phalanx (A, blue line) and the first distal phalanx (B, red line). This angle should normally measure < 10°. Radiographically, the hallux valgus interphalangeus angle is enlarged, indicating hallux valgus interphalangeus

Hallux valgus interphalangeus is a valgus deformity of the first digit at the interphalangeal joint, often associated with hallux valgus and bunion formation, though it can occur in isolation. The hallux valgus interphalangeus angle is measured between a line along the anatomic axis of the first distal phalanx and a line along the anatomic axis of the first proximal phalanx on a DP radiograph of the foot or great toe (Fig. 22). This angle should measure < 10° [65, 66] with greater measurements consistent with hallux valgus interphalangeus. It is useful in determining whether corrective surgery is indicated. In the setting of isolated interphalangeal valgus, a proximal phalangeal osteotomy (Akin Procedure) may be helpful in correcting the deformity, particularly in the setting of impingement on the second toe. However, when this is combined with hallux valgus, a more complex reconstruction, including bunionectomy and metatarsal osteotomy, may be needed [67].

##### First–second intermetatarsal angle

**Fig. 23:**
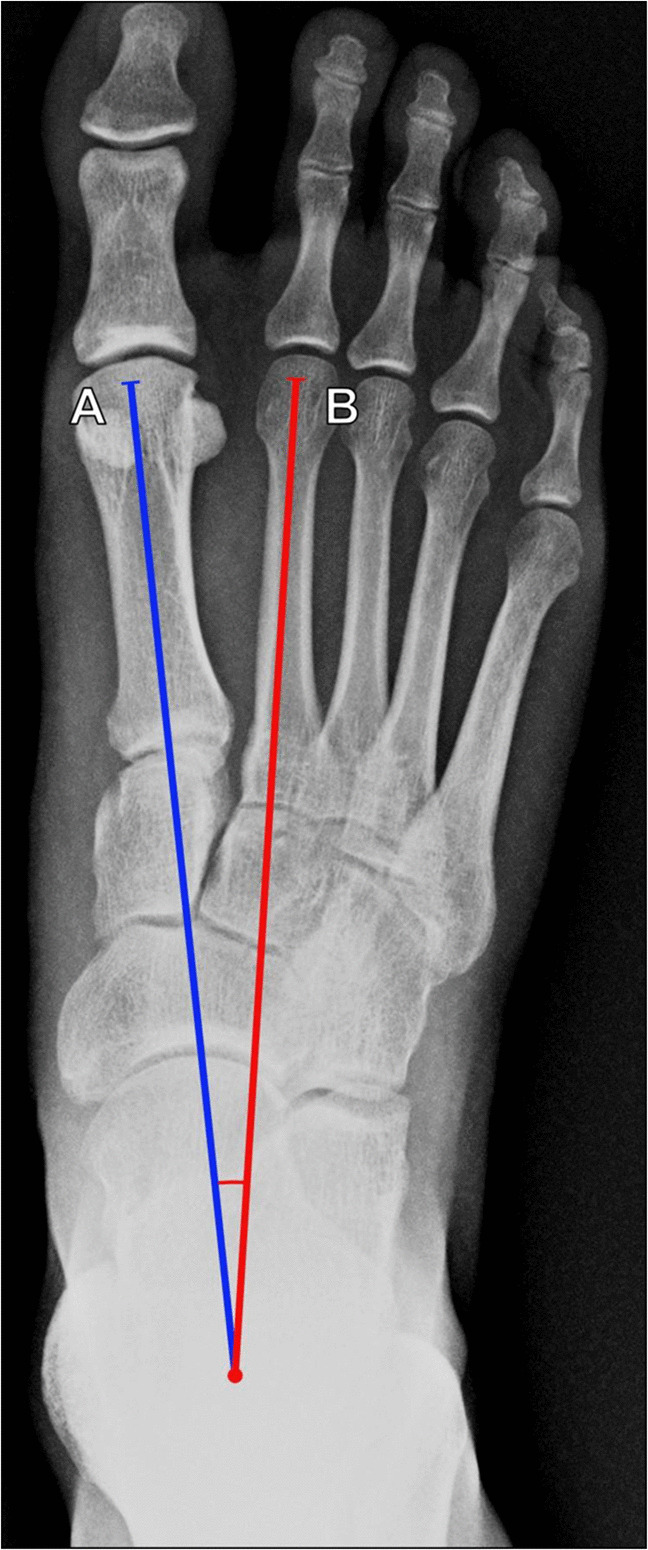
Measurement of first–second intermetatarsal angle on DP radiograph of the foot. The angle is formed between the long axis of the first metatarsal (A, blue line) and the long axis of the second metatarsal (B, red line). An angle ≥ 9° is associated with hallux valgus

The first–second intermetatarsal angle (1–2 IMA), sometimes shortened to the intermetatarsal angle, evaluates the severity of hallux abducto-valgus and first ray instability. Measured on DP weight bearing radiographs, the angle is formed by lines along the long axes of the first and second metatarsals (Fig. 23). The longitudinal axes should be centered on perpendicular transverse reference lines located 1 cm from the articular surfaces of the metatarsals [51]. If the second metatarsal is also medially deviated, a correction may need to be applied as the intermetatarsal angle will be artificially reduced [56]. A normal angle is < 9° [62]; an angle of 9°–11° indicates mild hallux valgus; 12°–17° is moderate; and ≥ 18° is severe [68]. There is a strong correlation between measurements made radiographically and on weightbearing CT [63, 64].

#### Less commonly used measurements to assess hallux valgus

##### Distal metatarsal articular angle

**Fig. 24:**
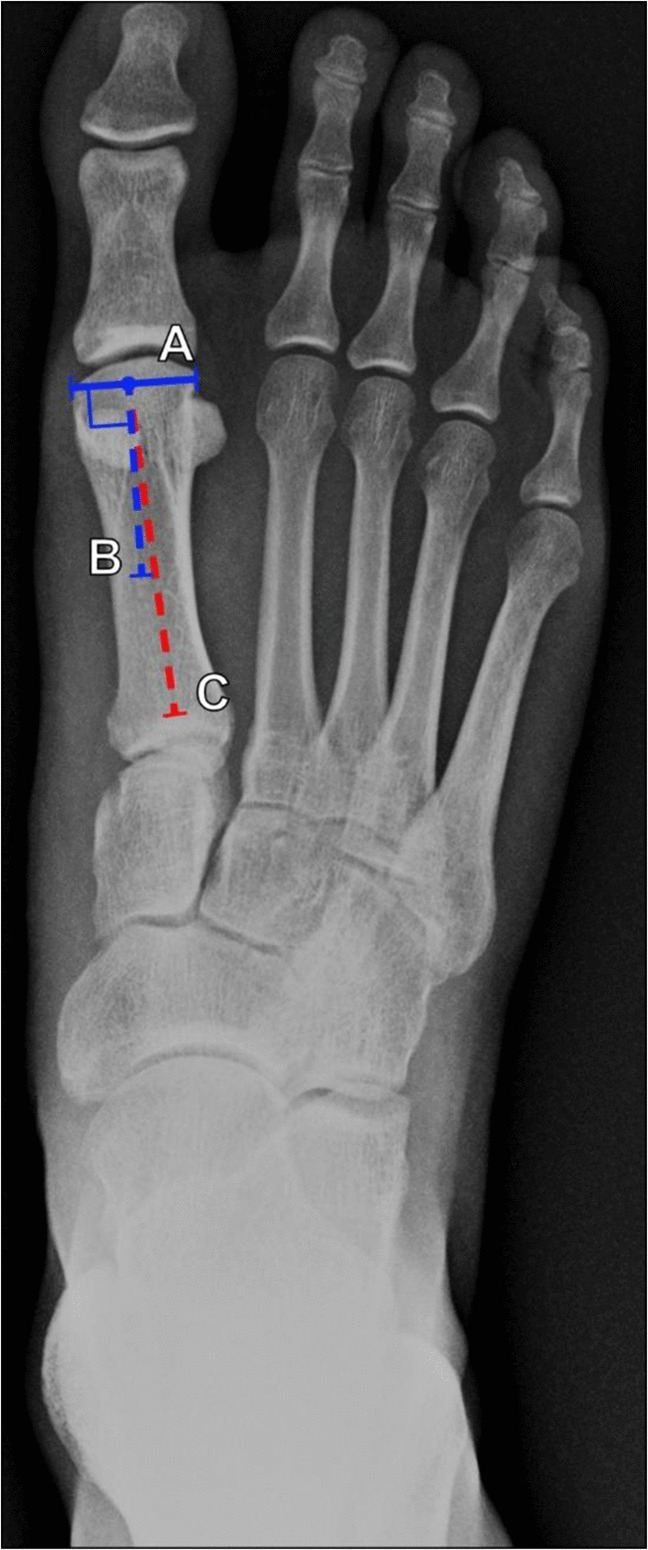
Measurement of the distal metatarsal articular angle (DMAA) on DP radiograph of the foot. Three lines are drawn: a line (A, solid blue line) from the medial to lateral margins of the first metatarsal head articular surface, a line (B, dashed blue line) perpendicular to line A, and a line (C, dashed red line) along the long axis of the first metatarsal. The angle is taken between line B and line C. Generally accepted normal values are ≤ 10°. While a higher DMAA does not necessarily indicate severity of the hallux valgus deformity, it does correlate with hallux valgus progression on longitudinal studies. In some patients, increased metatarsal head pronation may also result in abnormal measured DMAA values

The distal metatarsal articular angle (DMAA), sometimes called the proximal articular set angle, assesses the relationship of the articular surface of the first metatarsal relative to its shaft. The degree of first metatarsal pronation and plantarflexion may influence the measured value, which may make the measurement unreliable [69]; however, it is frequently used in literature and treatment planning. Patients with lower measured angles may respond well to a bunionectomy. However, those with higher values may require several osteotomies for appropriate correction. As described in Fig. 24, this angle is measured by drawing a line connecting the medial and lateral margins of the first metatarsal head articular surface and its perpendicular line, which forms one limb of the angle. The second limb is the metatarsal long axis. This measurement has alternatively been described as: a line drawn along the long axis of the first metatarsal with a line perpendicular to this that forms one limb of the angle; and a line connecting the medial and lateral margins of the first metatarsal head articular surface forming the second limb of the angle. Both methods of measurement yield the same results. It is theorized that changes in this angle may represent pronation of the first metatarsal head rather than a true deformity of the bone itself. The average normal angle was initially described as 6°. However, values are ≤ 10°generally accepted as normal. A higher DMAA does not necessarily indicate severity of the hallux valgus deformity, but it is correlated with progression of hallux valgus on longitudinal studies [1, 60, 69–72].

##### Sesamoid rotation angle

**Fig. 25:**
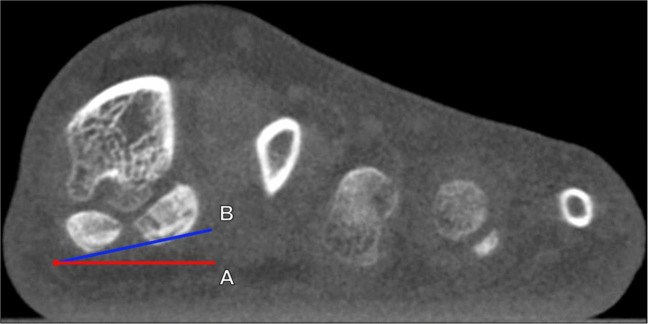
Measurement of hallux sesamoid rotation angle on coronal weightbearing CBCT of the foot. The angle is measured by drawing a line (A, red line) along the horizontal weightbearing surface and a line (B, blue line) connecting the inferior surfaces of the medial and lateral hallux sesamoids. Normal values range from 0° to 13°. An increasing angle represents worsening first MTP joint deformity and correlates with increased severity of hallux valgus

As the first metatarsal head deviates medially in hallux valgus, the angle of sesamoid rotation increases, and the degree of sesamoid displacement correlates highly with hallux valgus severity [73]. It can be assessed on weightbearing coronal reformatted images from CBCT or sesamoid tangential radiographs. On radiographs, this measurement reflects the angle between the horizontal weightbearing surface and a line connecting the plantar surfaces of the hallux sesamoid bones (Fig. 25) [74–78]. Measurements performed on CBCT or MR have shown excellent interrater reliability and correlation with hallux valgus angle, and the angle is formed by a line along the weight bearing surface of the forefoot connecting the skin beneath the first and fifth metatarsal heads, and a line drawn through the widest portion of the sesamoid apparatus. Normal values range from 0° to 13°. An increasing angle correlates with increased severity of hallux valgus and may require more complex corrective procedures, such as derotational osteotomy, to prevent recurrence of hallux valgus following surgery. Underestimation of the displacement can lead to incomplete surgical restoration leading to recurrence of the hallux valgus deformity [74].

##### First metatarsal pronation angle

**Fig. 26:**
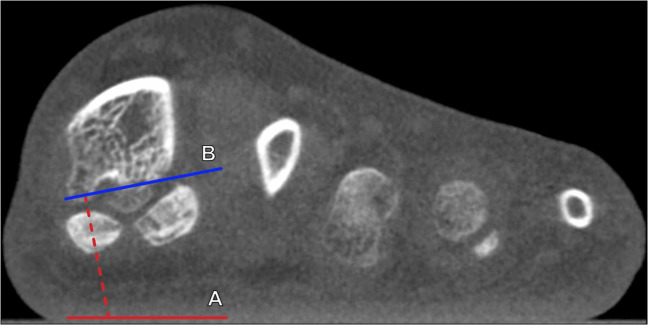
Measurement of the first metatarsal pronation angle on weightbearing CBCT of the foot. Measured on standing sesamoid tangential radiographs or a coronal weightbearing CBCT, the angle is formed between a horizontal line (A, red line) along the weightbearing surface of the foot and a line (B, blue line) from the lateral edge of the lateral hallux sesamoid sulcus to the medial edge of the medial hallux sesamoid sulcus. The first metatarsal pronation angle quantifies the degree of first metatarsal axial rotation along its long axis with lower (more negative) values indicating supination, and greater (more positive) values indicating greater pronation

The first metatarsal pronation angle is a measurement of the degree of first metatarsal axial rotation and is recognized as a clinically relevant component of hallux valgus deformity [79]. It is similar to the sesamoid rotation angle but is formed by line along the horizontal, weightbearing surface of foot and a line connecting the medial margin of the first metatarsal head medial hallux sesamoid fossa and the lateral margin of the lateral hallux sesamoid fossa (Fig. 26). This can be obtained on weightbearing coronal reformatted CBCT images or weightbearing sesamoid tangential radiographs, and there is excellent correlation between these modalities. Average normal values range from −4º to 12° with negative values representing first metatarsal supination. Greater first metatarsal pronation angles are associated with first MTP instability and may contribute to recurrent bunion formation after bunionectomy if not addressed during the initial surgical correction [80].

#### Congruency

In the context of the first MTP, congruency refers to the relative coverage of the first metatarsal head articular surface by the proximal phalangeal base. While the severity of hallux valgus is often determined by the degree of angulation, there are large differences in the choice of surgical method based on first MTP joint congruency. Congruent joints can be treated effectively with simpler procedures, such as an Akin Osteotomy, while incongruent joints may require more complex corrections for rotational or coronal plane deformities, including soft tissue release [81]. Congruency is also used postoperatively as an index to evaluate the effect of hallux valgus surgery [82].

This was historically qualitatively evaluated on weightbearing DP foot radiographs [83] based on subjective determination of whether the articular surfaces of the first metatarsophalangeal joint were parallel. Li et al. proposed the novel indices of metatarsophalangeal joint angle [84] and Congruence Index [85], which can be used to quantitatively evaluate first MTP joint congruency.

##### Metatarsophalangeal joint angle

**Fig. 27:**
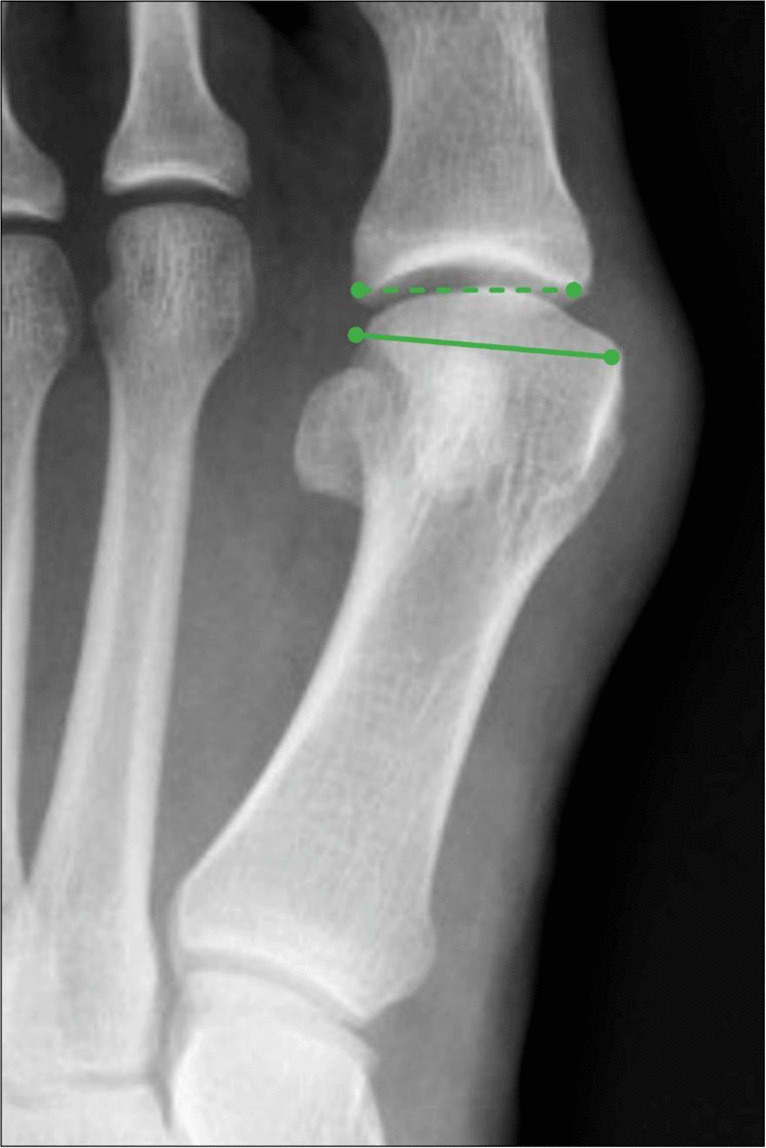
Measurement of the metatarsophalangeal joint angle on weightbearing DP radiograph of the foot. The angle is formed by a line (dashed line) connecting the medial and lateral margins of the proximal phalangeal base articular surface and a line (solid line) connecting the medial and lateral margins of the first metatarsal head articular surface. Normal angles are < 10° and indicate a congruent joint, and larger angles indicate greater metatarsophalangeal incongruency

The metatarsophalangeal joint angle is the angle formed by the first metatarsal head and first proximal phalangeal base articular surfaces. Normal angles are < 10° with increasing angles indicating incongruency (Fig. 27) [84].

##### Congruence Index

**Fig. 28:**
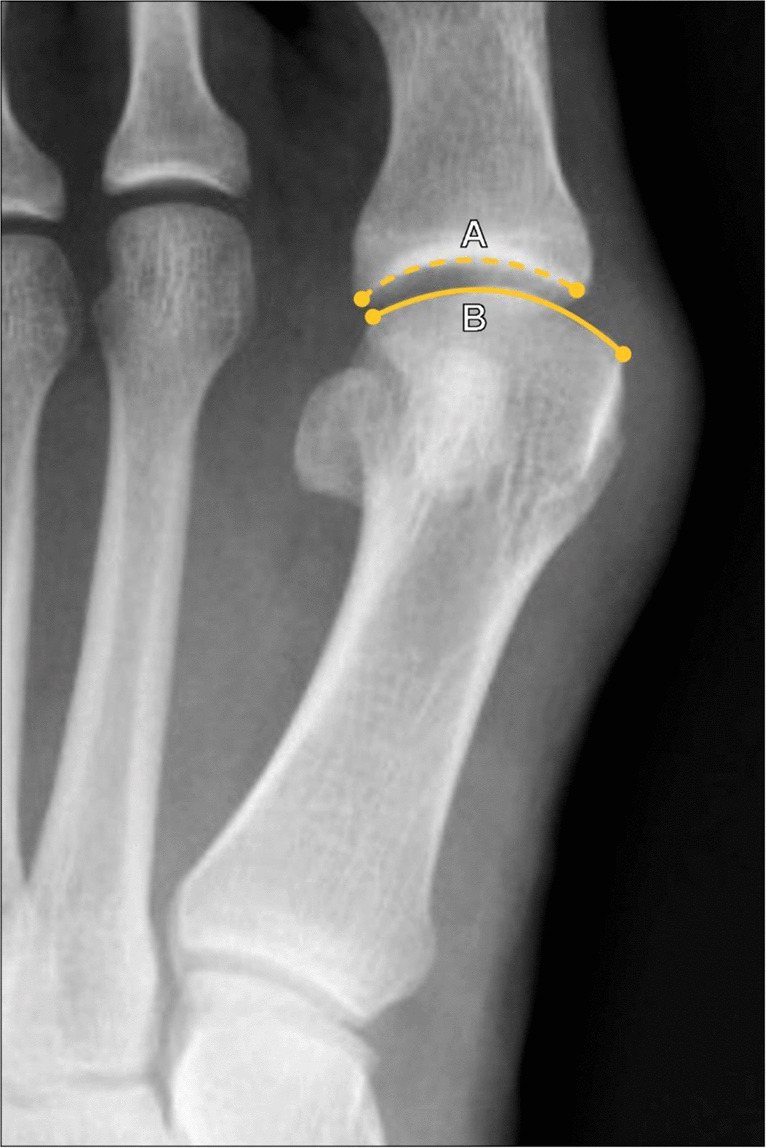
Measurement of Congruence Index on DP radiograph of the foot. The measurement indicates the degree of metatarsal head coverage by the proximal phalangeal base. To calculate the ratio, the portion of the first metatarsal head covered by the proximal phalanx (A, dashed line) is divided by the total length of the first metatarsal head articular surface (B, solid line) and multiplied by 100% to give a percentage. Normal values are > 76.5%, and lower values indicate greater incongruency

The Congruence Index reflects the degree of coverage of the first metatarsal articular surface by the first proximal phalangeal articular surface. As illustrated in Fig. 28, the length of the first metatarsal head articular surface covered by the proximal phalangeal base is divided by the length of the first metatarsal head articular surface, and the result is multiplied by 100% to give a percentage [85]. Normal values are > 76.5% with lower values indicating incongruency.

## Conclusion

As with the preceding two parts of this assessment of lower extremity measurements that focused on the hip and lower limb, as well as the knee, quantitative evaluation of the foot and ankle described in this review article represents a collection of the most commonly used angles, measures, and reference points. These radiographic measures provide the foundation for understanding foot and ankle deformities and are frequently used in when diagnosing foot and ankle abnormalities, pre-surgical planning, and post-surgical evaluation and management. As with imaging of the hip and knee, it is critical to understand the clinical indication for imaging and adhere to standardized patient positioning and techniques to produce reproducible measurements that can allow comparison on serial examinations.

## Data Availability

Not applicable.
